# Evolution of cancer stem cell lineage involving feedback regulation

**DOI:** 10.1371/journal.pone.0251481

**Published:** 2021-05-20

**Authors:** Iqra Batool, Naim Bajcinca

**Affiliations:** Faculty of Mechanical and Process Engineering, Technische Universität Kaiserslautern, Kaiserslautern, Rheinland Pfalz, Germany; Utrecht University, NETHERLANDS

## Abstract

Tumor emergence and progression is a complex phenomenon that assumes special molecular and cellular interactions. The hierarchical structuring and communication via feedback signaling of different cell types, which are categorized as the stem, progenitor, and differentiated cells in dependence of their maturity level, plays an important role. Under healthy conditions, these cells build a dynamical system that is responsible for facilitating the homeostatic regulation of the tissue. Generally, in this hierarchical setting, stem and progenitor cells are yet likely to undergo a mutation, when a cell divides into two daughter cells. This may lead to the development of abnormal characteristics, i.e. mutation in the cell, yielding an unrestrained number of cells. Therefore, the regulation of a stem cell’s proliferation and differentiation rate is crucial for maintaining the balance in the overall cell population. In this paper, a maturity based mathematical model with feedback regulation is formulated for healthy and mutated cell lineages. It is given in the form of coupled ordinary and partial differential equations. The focus is laid on the dynamical effects resulting from acquiring a mutation in the hierarchical structure of stem, progenitor and fully differentiated cells. Additionally, the effects of nonlinear feedback regulation from mature cells into both stem and progenitor cell populations have been inspected. The steady-state solutions of the model are derived analytically. Numerical simulations and results based on a finite volume scheme underpin various expected behavioral patterns of the homeostatic regulation and cancer evolution. For instance, it has been found that the mutated cells can experience significant growth even with a single somatic mutation, but under homeostatic regulation acquire a steady-state and thus, ensuing healthy cell population to either a steady-state or a lower cell concentration. Furthermore, the model behavior has been validated with different experimentally measured tumor values from the literature.

## Introduction

A tissue structure is comprised of various cell types arranged in a hierarchy according to specific characteristics, properties and functionalities. Typically, stem cells have the inherent property of indefinite self-renewal and differentiation into specialized cells [1]. Self-renewal in stem cells results in the production of the cells identical to the parent [2]. As sources of a lineage structure, stem cells produce progenitor cells via differentiation and their properties vary accordingly. For a given cell type, cell lineage has to undergo a number of maturity levels between the stem and differentiated cells. At the end of a cell line, the progenitor cells give rise to a mature cell population which does not possess the power to proliferate anymore, but can only experience apoptosis (the programmed cell death [3]). The specialised functions in the tissue are performed by mature cells, while the tissue homeostasis is preserved by regulating the ratio of stem cells’ self-renewal rate to differentiation. According to tumor stem cell hypothesis [4], cancer invasion and maintenance is driven by a small number of cells possessing the properties of stem cells. It has been observed that cancer initiating cells are characterized by high proliferative potential, capability to differentiate into diverse phenotypes and strength to escape apoptosis [4, 5]. In fact, these so-called “tumor-initiating cells” are stem cells that have acquired mutations [4], while the rest of the tumor cells are either mutated progenitor or differentiated cells. The latter can undergo apoptosis and are less likely responsible to invade and persist the tumor [6]. Therefore, it has been suggested to eradicate the cancer stem cells by treatment to completely eliminate the cancer [7]. This motivates particularly the study of stem cell dynamics and their role in the cancer evolution. In this sense, the present paper tends to develop a mathematical modeling framework, which is useful to predict the observed behavioral patterns of cancer evolution and, additionally, help in a purposeful impact by means of external inputs (e.g. radiation) which leads to mutation acquisition.

Tumor development results from acquiring mutations and escaping the enzyme-coded fixation process [8]. After acquiring a nonsense mutation, it can increase in number via cell division. Although not all mutations are harmful, certain mutations can contribute to malignant cell growth when acquired successively. While there exist various types of mutations, the ones which are crucial to cancer are characterized by enhanced proliferative potential, reduced apoptosis, genetic instability and reduced tumor suppression [5]. It has also been observed that typically one to ten mutations are required in a cell to revamp into a malignant one [5, 9, 10]. The mutated cells also possess a progeny, because these cells not only proliferate, but can also differentiate to successive cell types. In other words, there exists another hierarchical structure of mutated (i.e. cancer) cells besides the healthy one. Herein, the interesting aspect to study is the joint evolution of both progenies sharing the same environment.

The functionality of any multi-cellular organism as a whole depends greatly on the active feedback regulation process [11]. The loss of this homeostatic control escalates the growth of cells in the tissue which culminate in the advent of cancer. The precise nature of this feedback is not known [11]. In the literature, it is assumed that the mature cells secrete feedback signals which manipulate the stem cell’s division strength in order to maintain the balance between its self-renewal and differentiation rate [11]. The escalating growth of the cell population may approach the steady-state due to the effects induced by the feedback [12]. Various cell lineage frameworks have been introduced in the literature to investigate the dynamics of tissue regulation via feedback loop [11–13]. For a structural inspection of the feedback in a system consisting of two different cell lines with distinct properties, it is necessary to consider a model of each sub-population. The latter is based on the assumption that in every lineage, there exist a chain of maturation stages, which is sequentially arranged [14, 15].

Maturity represents a quantitative macroscopic measure which characterises cell differentiation. A variety of mathematical models have been formulated for explicit modeling of each cell subpopulation in a lineage using discrete [11, 12, 16–23] cell maturity representations. The discrete modeling paradigm assumes that maturation occurs only during the division of a cell, yielding a sequence of maturation stages. However, it is becoming evident that differentiation inherently displays continuous transitions. For instance, in neurogenesis cell differentiation without cell divisions are reported [24] and in haematopoiesis, stem cell lineage commitment is described to be a continuous process [25]. Besides, in some tissues, such as the mammary gland, different differentiation stages are not well-identified [26]. In addition to these experimental evidences, a rationale for considering continuous maturity representation is provided by the fact that differentiation is controlled by intracellular biochemical processes, which are continuous in time, at least when averaged over a large number of cells. Finally, modeling continuous maturation is exceptionally relevant considering the heterogeneity observed in various cancers (e.g. in breast cancer [27]) and thus necessitates a continuous maturity structured modeling approach. In the literature, continuous maturity structured models have been proposed in [28–30], where maturity is defined by a continuous variable which stands for the remaining proliferative potential of the cell and its capability to perform cellular functions. In [29], a continuous maturity structured model of granulopoiesis has been developed using partial differential equations (PDEs) for bone marrow granulocyte precursors and ordinary differential equation (ODE) for the blood granulocytes. The population of stem cells is assumed to be constant. The proliferation and mobilization rates along with apoptosis were modeled as functions of cell maturity. The scaled maturity level lies between zero and one. While the authors focused on the identification of the fastest mutation sequences leading to emergence of the cancer, the feedback regulation from the mature cells was entirely neglected. Due to the lack of regulation, such structures produce unbounded growth of cell populations only, and in particular can not predict steady-state evolution. On the other hand, in [30], the authors have used a similar maturity based continuous model along with additional stem cell dynamics in the form of an ODE model. The model is rather general and supports hierarchical structures of cell lineage. As opposed to [30], we assign a separate sub-population to mature cells and introduce the feedback homeostatic regulation therefrom, which has been neglected in both [29, 30]. Our model provides a generic framework to investigate the dynamics involved in the evolution of both normal and mutated cell populations under continuous maturation process and feedback regulation. The main motivation behind this model is to develop an insight into the process, while taking into account most relevant features of this multi-step process.

In the present paper, we consider the dynamical interaction of three different sub-populations: (i) the stem, (ii) progenitor and (iii) differentiated cells, while highlighting the effects of feedback regulation from the mature cell population. More specifically, we analyze the coupling of two progenies consisting of healthy and mutated cells, while our main interest lies in investigating the feedback regulation from the separately modeled dynamics of mature cell population into the stem and maturity structured progenitor cell populations. In our framework, the stem and differentiated cells are modeled using ODEs, assuming minimum and maximum maturity, respectively, while PDEs with continuous maturity distributions are used to predict the evolution of the progenitor cells. In particular, the differentiation rate is not assumed to be constant as in [30], it is rather considered to be a function of maturity. Although there exist several models with feedback regulation in the literature, to our best knowledge, this is a first attempt to cover the feedback regulation in a more generic framework of stem cell lineage with continuous maturity distribution along with the mutated cell lineage resulting from the mutation acquisition in healthy cells. Finally, it is also interesting to highlight that our mathematical model can predict the stem cell hypothesis, claiming that even a small number of mutated stem cells can invade the overall cell population.

The paper is organized as follows. In the sequel, we introduce our model of stem cell lineage for both healthy and mutated cell populations. Then, the steady-state solution are derived analytically in the following section. After that, we present the simulations of the developed model, its validation using the experimental data from the literature and the numerical scheme that has been implemented to find numerical solution. Finally, we conclude with the short outlook.

## Methods

### Mathematical model

**Fig 1:**
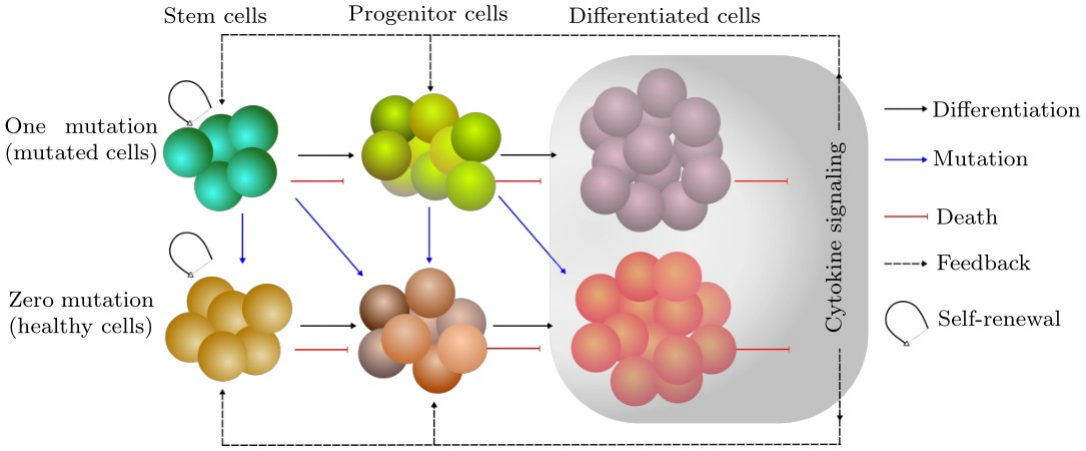
Schematics of the model. Co-evolution of normal and mutated cells in the presence of feedback regulation. The model captures the evolution of stem cell lineages with zero and one mutation. Stem cells can self-renew, differentiate and undergo apoptosis. The resulting progeny continues to proliferate until terminally differentiates into mature cells. Note that, during the division of a cell, there is a probability of getting a mutation. The feedback originates from the mature cells and thus regulates the self-renewal and proliferation rate of stem and progenitor cells, respectively.

The mathematical model of the stem cell line is rather complex as the cells vary continuously in course of maturation with time. In the present paper, instead of considering the evolution of net cell population, we split it into different sub-populations to account for their specific dynamics, as depicted in Fig 1. The very initial cell state, i.e., stem cells, has the potential to stay undifferentiated and not to divide frequently under the conditions of homeostatic regulation [1, 31, 32]. As a middle stage in the cell evolution from stem cells all the way to full differentiation, we discriminate the progenitor cells, which undergo proliferation at relatively high rates and give rise to the population of fully mature cells. In the process of maturation, the proliferative potential and mortality rate of progenitor cells vary drastically until the terminal differentiation. To capture these dynamical effects, it is necessary to consider the maturity distribution of progenitor cells, which is mathematically described by means of PDEs. The last transition stage in the cell line from progenitor cells refers to fully mature cells that are specialized to perform their functions in the respective tissue without further division, and undergo apoptosis after a short span of life.

The schematics of our model in Fig 1 depicts the possible interactions between subsequent cell types ensuing from symmetric/asymmetric self-renewal, mutation, differentiation and apoptosis. The two parallel cell lines refer to the healthy and mutated cells (perhaps cancer, if cells acquire a lethal mutation) with zero and one mutation, respectively. Notice that, we consider only one mutation to keep the model simple for investigation. The model can be scaled up easily to the acquisition of multiple mutations. The potential for self-renewal is labeled as the property only for the stem cells in both healthy and mutated states, while the differentiation of cells is undertaken by both stem cell and progenitor cell populations. However, apoptosis can occur at all transition states with a certain rate. Since the number of cells increases with each step of maturation [33], the evolution scheme of all cell states as described above may lead to abnormal growth tending towards an unbounded number of cells. To avoid such unrestrained behavior of cell growth, one has to introduce feedback regulation. The modeling scheme in Fig 1 enables investigation of the dynamical behavior (i.e., evolution and control) of the overall cell population with and without feedback homeostatic regulation. The discrete compartmental setting of the model facilitates the implementation and withdrawal of the feedback signals into and from the different transition states, respectively.

In the sequel, we explain the governing model equations for the cell lineage dynamics of healthy and mutated cells. Thereby, *C*_0_(*t*) and *C*_1_(*t*) refer to the number of stem cells with zero and one mutation, respectively. Similarly, *P*_0_(*x*, *t*) and *P*_1_(*x*, *t*) correspond to the progenitor cells with zero and one mutation, respectively, while *M*_0_(*t*) and *M*_1_(*t*) refer to the number of fully differentiated healthy and mutated cell populations. As the cells in the compartment of the stem and mature cells are assumed to behave alike, one can infer a modeling scheme in the form of ODEs. On the other hand, the healthy and mutated progenitor cells require a property space, with cell maturity as a property variable. Since all the intermediate transitioning cell states between stem and fully differentiated cells are modeled as progenitor cells, the cells therein continuously differentiate to higher maturity states. Thus, the progenitor cells in both healthy and mutated states require PDEs.

#### Stem cell population

Stem cells are assumed to possess zero maturity level and they define the boundary conditions for the progenitor cell population at minimum maturity. The primary characteristics of stem cells responsible for their evolution are self-renewal and differentiation. The self-renewal can occur in two different ways, symmetrically or asymmetrically. Either way, there is a probability of acquiring a mutation during the division process, this yields an influx into the mutated stem cell population. On the other hand, differentiation of stem cells without mutation acquisition results in healthy progenitor cells, and those with a mutation influence to mutated progenitor cells. The stem cell population increases by symmetric self-renewal only, whereas the other mechanisms, e.g., mutation acquisition and differentiation, cause a decrement. The dynamical behavior of the stem cells is then described by the following mathematical expressions

$$\frac{d}{dt}C_{0}\left(t\right)=\left[\left(1-2m\right)\alpha_{S_{0}}\left(s\right)-m\alpha_{A_{0}}\left(s\right)-\alpha_{D_{0}}\left(s\right)-\delta_{C_{0}}\right]k_{0}C_{0}\left(t\right),\;\;$$ (1)

$$\frac{d}{dt}C_{1}\left(t\right)=\left[\alpha_{S_{1}}\left(s\right)-\alpha_{D_{1}}\left(s\right)-\delta_{C_{1}}\right]k_{1}C_{1}\left(t\right)+\left[2m\alpha_{S_{0}}\left(s\right)+m\alpha_{A_{0}}\left(s\right)\right]k_{0}C_{0}\left(t\right).$$ (2)

The initial conditions of healthy and mutated stems cells are *C*_0_(0) = *c*_0_ and *C*_1_(0) = *c*_1_, respectively. In the above equations, *k*_0_ and *k*_1_ are the proliferation rates of stem cell with zero and one mutation, respectively. The first term on the right-hand side in Eq (1) refers to symmetric self-renewal with probability $\alpha_{S_{0}}$, which results either in a decrement in the stem cell population by one, if the stem cells acquire a mutation with rate *m*, i.e. $-\alpha_{S_{0}}\left(s\right)mk_{0}C_{0}$, or increase the pool by one in case of no mutation, i.e. $\left(1-m\right)\alpha_{S_{0}}\left(s\right)k_{0}C_{0}$. The second term represents an asymmetric self-renewal of stem cells with probability $\alpha_{A_{0}}$ in which the stem cells decrease by one, while asymmetrically self-renewing and acquiring a mutation. The third term represents the differentiation of stem cells with probability $\alpha_{D_{0}}$, which is always followed by a decrement in stem cell population by one. The resulting progeny from the differentiation of cells will influx into the progenitor cell population. Note that the feedback signal *s* has been introduced into the stem cell probability of self-renewal (symmetric/asymmetric) and differentiation to maintain tissue homeostasis. This feedback signal is determined by the mature cell population, as shown below in Eq (12). Finally, the fourth term describes the death of stem cells with a rate of $\delta_{C_{0}}$, which reduces the stem cell population by one.

In Eq (2), the first three terms in a square bracket on the right-hand side describe the symmetric self-renewal, differentiation and death of mutated stem cells *C*_1_ with the probability of $\alpha_{S_{1}}$ $\alpha_{D_{1}}$, and $\delta_{C_{1}}$, respectively. The last two terms (in the second square bracket) correspond to the influx from healthy stem cell population *C*_0_ as a consequence of mutations acquired during symmetric and asymmetric self-renewal, Eq (1).

#### Progenitor cell population

The maturity distribution of progenitor cells represented by *P*_0_(*x*, *t*) and *P*_1_(*x*, *t*) constitutes of all maturity stages between stem and mature cell populations with *x* as maturity variable. Obviously, $\int_{x_{1}}^{x_{2}}P_{i}\left(x,t\right)dx$, *i* = 0, 1, is equal to the number of cells between maturity *x*_1_ and *x*_2_. The governing equations and the initial conditions for normal and mutated progenitor cells read:

$$\partial_{t}P_{0}\left(x,t\right)+\partial_{x}\left[g_{0}\left(x\right)P_{0}\left(x,t\right)\right]=\left[\left(1-2m^{\prime }\right)\beta_{0}\left(x,s\right)-\mu_{0}\left(x\right)\right]P_{0}\left(x,t\right),\;\;$$ (3)

$$\partial_{t}P_{1}\left(x,t\right)+\partial_{x}\left[g_{1}\left(x\right)P_{1}\left(x,t\right)\right]=\left[\beta_{1}\left(x,s\right)-\mu_{1}\left(x\right)\right]P_{1}\left(x,t\right)+2m^{\prime }\beta_{0}\left(x,s\right)P_{0}\left(x,t\right),\;\;$$ (4)

with initial conditions *P*_0_(*x*, 0) = *p*_0_(*x*), *P*_1_(*x*, 0) = *p*_1_(*x*) and boundary conditions

$$g_{0}\left(0\right)P_{0}\left(0,t\right)=\left[2\left(1-m\right)\alpha_{D_{0}}\left(s\right)+\left(1-m\right)\alpha_{A_{0}}\left(s\right)\right]k_{0}C_{0}\left(t\right),$$ (5)

$$g_{1}\left(0\right)P_{1}\left(0,t\right)=\left[2\alpha_{D_{1}}\left(s\right)+\alpha_{A_{1}}\left(s\right)\right]k_{1}C_{1}\left(t\right)+\left[2m\alpha_{D_{0}}\left(s\right)+m\alpha_{A_{0}}\left(s\right)\right]k_{0}C_{0}\left(t\right),$$ (6)

for *t* > 0.

**Fig 2:**
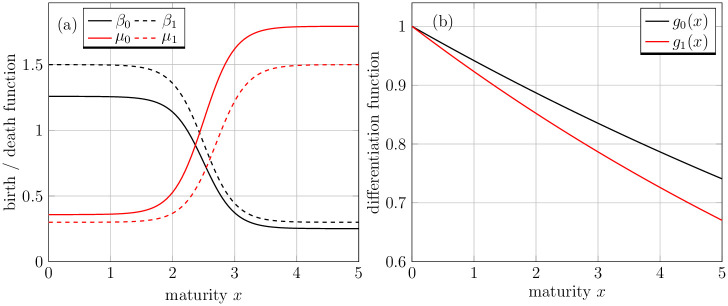
Birth, death and differentiation functions for progenitor cells. (a) Birth and death functions for both, healthy and mutated progenitor cell populations. The solid black and red lines represent the birth and death rate of healthy progenitor cell population, respectively, whereas the dotted black and and red lines depict the birth and death rate of mutated progenitor cells. (b) Differentiation function of healthy and mutated progenitor cell populations represented by black and red line, respectively.

The functions *g*_0_(*x*) and *g*_1_(*x*) stand for the differentiation rate of progenitor cells with zero and one mutation, respectively. On the right-hand side of Eq (3), the first and second terms in the square bracket represent the birth and loss of progenitor cells due to a mutation with the rate *m*′. The progenitor cells are assumed to acquire one mutation at a time. The proliferation rates *β*_0_(*x*, *s*) and *β*_1_(*x*, *s*) of healthy and mutated progenitor cells depend on the maturity level and tend to zero as the cells achieve the higher maturity level [34]. The third term describes the apoptosis of progenitor cells *P*_0_(*x*, *t*) with maturity dependent death rate *μ*_0_(*x*) and generally it gets higher as the cell matures. On the right-hand side of Eq (4), the first two terms in a square bracket represent the proliferation and death of the mutated progenitor cells with the rate *β*_1_(*x*, *s*) and *μ*_1_(*x*), respectively. The last term represents the influx from the healthy progenitor cells via mutation. Note that, the proliferation potential and rate of apoptosis for the progenitor cells is defined as function of maturity. The early progenitor cells have higher proliferation potential as compared to the late progenitor cells. On the other hand, as mentioned earlier the death rate of progenitor cells is meager for early progenitor cells and increases after differentiating to a certain maturity level [35, 36], see Fig 2(a). Although this does not hold for all cell types but true for of haemopoietic cells. There are many choices which can be suitable for proliferation and death rates of progenitor cells. Here, we borrow from [30] the following functional forms for *β*_*i*_ and *μ*_*i*_,

$$\beta_{i}\left(x,s\right)=-\frac{1}{2}b_{i}\left(s\right)\;\text{tanh}\;\left(\rho_{\beta_{i}}\left(x-\omega_{\beta_{i}}\right)\right)+\frac{1}{2}b_{i}\left(s\right),$$ (7)

$$\mu_{i}\left(x\right)=\frac{1}{2}d_{i}\;\text{tanh}\;\left(\rho_{\mu_{i}}\left(x-\omega_{\mu_{i}}\right)\right)+\frac{1}{2}d_{i},\;\;$$ (8)

where *i* = 0, 1, and *b*_*i*_ and *d*_*i*_ represent the maximum rate of proliferation and apoptosis, respectively. Furthermore, $\omega_{\beta_{i}}$ represent the maturity level at which the progenitor cells proliferate at half of the maximum rate and $\rho_{\beta_{i}}$ refers to the steepness of the proliferation switch. Similarly, the maturity at which progenitor cells die at half of the maximum rate is $\omega_{\mu_{i}}$ and the steepness of the switch is $\rho_{\mu_{i}}$. Here, the feedback signal *s* is introduced in the proliferation rate of progenitor cells. The behavior of the functional forms of *β*_*i*_ (for a fixed value of feedback, i.e., *s* = 1) and *μ*_*i*_ are depicted in Fig 2(a).

Next, we introduce the differentiation function *g*_*i*_(*x*) which describes the rate at which the cells mature. It is a strictly positive and continuously differentiable function. In the pool of progenitor cells, continuous differentiation takes place alongside cellular division (maturation process). In maturity-time representation, mitosis takes place at same maturity levels [37]. From the modeling viewpoint, it means that in an infinitesimal time interval (*t*, *t* + d*t*), a cell with maturity *x* either matures to level *x* + d*x* with probability *g*(*x*)d*t* or divides into two daughter cells with probability *β*(*x*, *s*)d*t*. The progenitor cell population is heterogeneous with respect to cell maturation velocities [37] and typically, the maturity rate decreases along with an increasing maturity level. In order to define *g*(*x*), we fix the maximum maturation velocity equal to one then we use a monotonically decreasing function of the following functional form *g*_*i*_(*x*) = exp(−*h*_*i*_ *x*), *i* = 0, 1 in our model. Here, the differentiation functions *g*_*i*_(*x*) are bounded between 0 and 1, thus the parameters *h*_*i*_ are to be selected in such a way that *g*_*i*_(*x*) should not get near zero within the specified range of maturity variable, i.e., *x*_min_ ≤ *x* ≤ *x*_max_, see Fig 2(b). We assume lower differentiation potential for mutated cells because poor cellular differentiation is one of the important traits of cancer [38, 39].

During a division process, progenitor cells can also undergo a mutation. In this model, the healthy progenitor cells *P*_0_(*x*, *t*) acquire a mutation with a mutation rate *m*′ to either proliferate into a mutated progenitor cell population *P*_1_(*x*, *t*) or to differentiate into a mutated differentiated cell population *M*_1_(*x*, *t*).

#### Mature cell population

The mature cell population is constituted by all the cells that attain the maximum maturity level, i.e. x*. Here, maximum maturity is assumed to be same for both normal and mutated cells. Normally, cancers are graded as ‘low-differentiated’ and ‘well-differentiated’ cancers based on the level of maturation of the cells in an organ where the cancer arises. This implies that mutated cells are as mature as normal cells in ‘well-differentiated’ cancers. However, mutated cells may remain immature sometimes if mutations are taking place in early phases of maturation because mutated cells grow rapidly and divide before cells are fully mature. Nevertheless, it is plausible to assume that also the mutates cells can achieve the maximum maturation level. Mature cells do not possess any proliferating potential and only undergo apoptosis after a particular time. Therefore, mature cells cannot acquire any additional mutation and are specialized to perform their assigned functions in the tissue. The following equations describe the healthy and mutated density of mature cells represented by *M*_0_(*t*) and *M*_1_(*t*), respectively:

$$\frac{dM_{0}}{dt}=g_{0}\left(x^{*}\right)P_{0}\left(x^{*},t\right)-\delta_{M_{0}}M_{0},\;M_{0}\left(0\right)=m_{0},$$ (9)

$$\frac{dM_{1}}{dt}=g_{1}\left(x^{*}\right)P_{1}\left(x^{*},t\right)+2m^{\prime }g_{0}\left(x^{*}\right)P_{0}\left(x^{*},t\right)-\delta_{M_{1}}M_{1},\;M_{1}\left(0\right)=m_{1}.$$ (10)

In Eq (9), the first term on the right-hand side describes the inflow into healthy mature cells via terminal differentiation of progenitor cells with maturity rate *g*_0_(x*), while the second term defines the apoptosis of the mature cells with a rate of $\delta_{M_{0}}$. The first term on the right-hand side of Eq (10) is the influx from fully differentiated mutated progenitor cells, and the second term represents the influx from healthy progenitor cells due to an acquired mutation. The last term involving the rate $\delta_{M_{1}}$ refers to the death of mutated mature cell population.

#### Feedback regulation

In the signaling mechanism among the cells, the growth response is modulated by cytokine proteins along with other proliferation regulating factors [11]. Cytokines bind to their specific membrane-associated receptors which results in the activation of signal transduction pathways [40]. Studies have shown that in order to maintain the number of cells in balance, these signals have to depend on the mature cell population [41, 42]. The dynamics of cytokine signaling molecules *S* can be described by an ODE as: $\dot{S}=\upsilon -\delta_{S}S-\gamma SM,$ where *υ* is the maximum secretion rate of cytokine signals, *δ*_*S*_ represents the natural decrement of the signals *S*, and *γ* is the rate by which the total mature cell population *M* = *M*_0_ + *M*_1_ (consisting of both healthy and mutated mature cells) regulate the cytokine signals. Substituting *s* = (*δ*_*S*_/*υ*)*S* and *k*_*s*_ = *γ*/*δ*_*S*_, the above equation turns into

$$\begin{array}{c} \dot{s}=\delta_{S}\left(1-s-k_{s}sM\right). \end{array}$$ (11)

Since the cytokine signals are typically secreted at a higher rate than that of proliferation and differentiation of the cells, these drift quickly to a steady state. Hence, using quasi-steady state approximation, the equilibrium state for the feedback signal intensity

$$\begin{array}{c} s=\frac{1}{1+k_{s}M} \end{array}$$ (12)

follows from Eq (11). This shows that in the absence of mature cell population, the signal intensity is maximal, i.e., *s* = 1, and it drops to a minimum with a significant increase in the mature cell population. The nature of this signal can be understood as a controlling parameter that identifies the need for proliferation based on how many cells are present in the vicinity. These signals get weaker and weaker with a larger number of cells, i.e., there are not enough resources critical for the division process.

The probabilities $\alpha_{S_{i}}\left(s\right)$, $\alpha_{A_{i}}\left(s\right)$ and $\alpha_{D_{i}}\left(s\right)$ in Eqs (1) and (2) and the maximum birth rates *b*_*i*_(*s*) in the birth functions *β*_*i*_(*x*, *s*) of progenitor cells in Eqs (3) and (4) are assumed to change linearly in *s*, cf. Eq (7), given that slopes are positive which leads to the following linear forms $\alpha_{S_{i}}\left(s\right)={\bar{\alpha }}_{S_{i}}s$, $\alpha_{A_{i}}\left(s\right)={\bar{\alpha }}_{A_{i}}s$, $\alpha_{D_{i}}\left(s\right)={\bar{\alpha }}_{D_{i}}s$ and $b_{i}\left(s\right)={\bar{b}}_{i}s$, where ${\bar{\alpha }}_{S_{i}}$, ${\bar{\alpha }}_{A_{i}}$, ${\bar{\alpha }}_{D_{i}}$ and ${\bar{b}}_{i}$ represent maximum symmetric self-renewal, maximum asymmetric self-renewal, maximum differentiation of stem cell and maximum birth rate of progenitor cells, respectively.

## Results

### Steady-state solutions

In this section, we derive the steady-state solutions of our governing Eqs (1)–(10). For the sake of convenience, we hereby simplify the notation, reading

$$\frac{d}{dt}C_{0}\left(t\right)=\gamma_{00}\left(s\right)C_{0}\left(t\right),\;\;C_{0}\left(0\right)=c_{0}$$ (13)

$$\frac{d}{dt}C_{1}\left(t\right)=\gamma_{10}\left(s\right)C_{1}\left(t\right)+\gamma_{11}\left(s\right)C_{0}\left(t\right),\;\;C_{1}\left(0\right)=c_{1},$$ (14)

for the populations of healthy and mutated cells, respectively, with *γ*_00_, *γ*_10_ and *γ*_11_ defined as

$$\gamma_{00}\left(s\right)≔\left[\left(1-2m\right)\alpha_{S_{0}}\left(s\right)-m\alpha_{A_{0}}\left(s\right)-\alpha_{D_{0}}\left(s\right)-\delta_{C_{0}}\right]k_{0}$$ (15)

$$\gamma_{10}\left(s\right)≔\left[\alpha_{S_{1}}\left(s\right)-\alpha_{D_{1}}\left(s\right)-\delta_{C_{1}}\right]k_{1},\;\;\gamma_{11}\left(s\right)=\left[2m\alpha_{S_{0}}\left(s\right)+m\alpha_{A_{0}}\left(s\right)\right]k_{0}.$$ (16)

In a similar manner, the PDEs that describe the progenitor cells read:

$$\partial_{t}P_{0}\left(x,t\right)+\partial_{x}\left[g_{0}\left(x\right)P_{0}\left(x,t\right)\right]=\gamma_{0}\left(x,s\right)P_{0}\left(x,t\right),$$ (17)

$$\partial_{t}P_{1}\left(x,t\right)+\partial_{x}\left[g_{1}\left(x\right)P_{1}\left(x,t\right)\right]=\gamma_{1}\left(x,s\right)P_{1}\left(x,t\right)+2m^{\prime }\beta_{0}\left(x,s\right)P_{0}\left(x,t\right),$$ (18)

where *γ*_0_(*x*, *s*) and *γ*_1_(*x*, *s*) are given by

$$\begin{array}{c} \gamma_{0}\left(x,s\right)≔\left(1-2m^{\prime }\right)\beta_{0}\left(x,s\right)-\mu_{0}\left(x\right),\;\;\gamma_{1}\left(x,s\right)≔\beta_{1}\left(x,s\right)-\mu_{1}\left(x\right). \end{array}$$ (19)

Finally, the equations of mature cell population stay same as before in Eqs (9) and (10). In the sequel, we assume the following conditions:

$$\begin{array}{c} \begin{array}{c} c_{0},c_{1},m_{0},m_{1}\in R_{\geq 0},\;\;p_{0},\;p_{1}:\left[0,x^{*}\right]\to R_{\geq 0}\\ g_{0_{x}},g_{1_{x}}\in L^{\infty }\left(\left[0,x^{*}\right]\right)\\ \beta_{0}\left(x,s\right),\;\;\beta_{1}\left(x,s\right)\in L^{\infty }\left(\left[0,x^{*}\right]\times R\right),\;\;\mu_{0}\left(x\right),\;\;\mu_{1}\left(x\right)\in L^{\infty }\left(\left[0,x^{*}\right]\right)\\ \gamma_{00}\left(s\right)≔\gamma_{00}\left(M\right)\;\text{is}\;\text{a}\;\text{decreasing}\;\text{function,}\;\text{i.e.,}\;\gamma_{00}\left(+\infty \right)<0. \end{array}\} \end{array}$$ (20)

To address the question of the existence of any steady-state under a homeostatic regulation, we need to solve the following system of equations, for the steady-state unknowns ${\bar{C}}_{0}$, ${\bar{C}}_{1}$, ${\bar{P}}_{0}$, ${\bar{P}}_{1}$, ${\bar{M}}_{0}$ and ${\bar{M}}_{1}$:

$$\gamma_{00}\left(\bar{M}\right){\bar{C}}_{0}=0$$ (21)

$$\gamma_{10}\left(\bar{M}\right){\bar{C}}_{1}+\gamma_{11}\left(\bar{M}\right){\bar{C}}_{0}=0$$ (22)

$$\frac{d}{dx}\left[g_{0}\left(x\right){\bar{P}}_{0}\left(x\right)\right]={\bar{\gamma }}_{0}\left(x\right){\bar{P}}_{0}\left(x\right)$$ (23)

$$\frac{d}{dx}\left[g_{1}\left(x\right){\bar{P}}_{1}\left(x\right)\right]={\bar{\gamma }}_{1}\left(x\right){\bar{P}}_{1}\left(x\right)+2m^{\prime }{\bar{\beta }}_{0}\left(x\right){\bar{P}}_{0}\left(x\right)$$ (24)

$$g_{0}\left(x^{*}\right){\bar{P}}_{0}\left(x^{*}\right)-\delta_{M_{0}}{\bar{M}}_{0}=0$$ (25)

$$\;g_{1}\left(x^{*}\right){\bar{P}}_{1}\left(x^{*}\right)+2m^{\prime }g_{0}\left(x^{*}\right){\bar{P}}_{0}\left(x^{*}\right)-\delta_{M_{1}}{\bar{M}}_{1}=0,$$ (26)

with ${\bar{\gamma }}_{0}\left(x\right)≔\gamma_{0}\left(x,\bar{M}\right)$, ${\bar{\gamma }}_{1}\left(x\right)≔\gamma_{1}\left(x,\bar{M}\right)$ and ${\bar{\beta }}_{0}\left(x\right)=\beta_{0}\left(x,\bar{M}\right)$ and the boundary conditions given as

$$g_{0}\left(0\right){\bar{P}}_{0}\left(0\right)=\left(1-m\right)\left[2\alpha_{D_{0}}\left(\bar{M}\right)+\alpha_{A_{0}}\left(\bar{M}\right)\right]k_{0}{\bar{C}}_{0}$$ (27)

$$g_{1}\left(0\right){\bar{P}}_{1}\left(0\right)=\left[2\alpha_{D_{1}}\left(\bar{M}\right)+\alpha_{A_{1}}\left(\bar{M}\right)\right]k_{1}{\bar{C}}_{1}+m\left[2\alpha_{D_{0}}\left(\bar{M}\right)+\alpha_{A_{0}}\left(\bar{M}\right)\right]k_{0}{\bar{C}}_{0}.$$ (28)

The trivial steady-state, i.e., ${\bar{C}}_{0}=0$, ${\bar{C}}_{1}=0$, ${\bar{P}}_{0}=0$, ${\bar{P}}_{1}=0$, ${\bar{M}}_{0}=0$, ${\bar{M}}_{1}=0$ is evident from Eqs (21)–(26). However, the system also admits a non-trivial steady-state under the assumption *γ*_00_(0) > 0. In this case, from Eq (21) we get immediately

$$\begin{array}{c} \gamma_{00}\left(\bar{M}\right)=0. \end{array}$$ (29)

Now, using Eq (15) in the above relation, we obtain:

$$\begin{array}{c} \left[\left(1-2m\right)\alpha_{S_{0}}\left(\bar{M}\right)-m\alpha_{A_{0}}\left(\bar{M}\right)-\alpha_{D_{0}}\left(\bar{M}\right)-\delta_{C_{0}}\right]k_{0}=0, \end{array}$$ (30)

where the probabilities $\alpha_{S_{0}}\left(\bar{M}\right)$, $\alpha_{A_{0}}\left(\bar{M}\right)$, and $\alpha_{D_{0}}\left(\bar{M}\right)$ are defined as

$$\begin{array}{c} \alpha_{S_{0}}\left(\bar{M}\right)=\frac{{\bar{\alpha }}_{S_{0}}}{1+k_{s}\bar{M}},\;\;\alpha_{A_{0}}\left(\bar{M}\right)=\frac{{\bar{\alpha }}_{A_{0}}}{1+k_{s}\bar{M}},\;\;\alpha_{D_{0}}\left(\bar{M}\right)=\frac{{\bar{\alpha }}_{D_{0}}}{1+k_{s}\bar{M}}, \end{array}$$ (31)

and ${\bar{\alpha }}_{S_{0}}$, ${\bar{\alpha }}_{A_{0}},{\bar{\alpha }}_{D_{0}}\in R_{>0}$. By employing the above relations in Eq (30), we derive the relation for $\bar{M}$, which is:

$$\begin{array}{c} \bar{M}=\frac{1}{k_{s}\delta_{C_{0}}}\left[\left(1-2m\right){\bar{\alpha }}_{S_{0}}-m{\bar{\alpha }}_{A_{0}}-{\bar{\alpha }}_{D_{0}}-\delta_{C_{0}}\right]. \end{array}$$ (32)

Next, to solve the ODEs (23) and (24) for progenitor cells, we compute the boundary conditions at the final maturity, i.e., *x* = x* from the Eqs (25) and (26)

$$\begin{array}{c} \bar{P_{0}}\left(x^{*}\right)=\frac{\delta_{M_{0}}{\bar{M}}_{0}}{g_{0}\left(x^{*}\right)},\;\;{\bar{P}}_{1}\left(x^{*}\right)=-\frac{2m^{\prime }\delta_{M_{0}}{\bar{M}}_{0}}{g_{1}\left(x^{*}\right)}+\frac{\delta_{M_{1}}{\bar{M}}_{1}}{g_{1}\left(x^{*}\right)}. \end{array}$$ (33)

The steady-state values result by solving the ODEs (23) and (24) for the healthy and mutated progenitor cells ${\bar{P}}_{0}\left(x\right)$ and ${\bar{P}}_{1}\left(x\right)$:

$${\bar{P}}_{0}\left(x\right)=\frac{\delta_{M_{0}}{\bar{M}}_{0}}{g_{0}\left(x\right)}e^{f_{0}\left(x\right)},$$ (34)

$${\bar{P}}_{1}\left(x\right)=\frac{e^{f_{1}\left(x\right)}}{g_{1}\left(x\right)}[2m^{\prime }\int_{x}^{x^{*}}e^{-f_{1}\left(x\right)}{\bar{\beta }}_{0}\left(x\right){\bar{P}}_{0}\left(x\right)dx-2m^{\prime }\delta_{M_{0}}{\bar{M}}_{0}+\delta_{M_{1}}{\bar{M}}_{1}],$$ (35)

with

$$\begin{array}{c} f_{0}\left(x\right)≔\int_{x}^{x^{*}}\frac{{\bar{\gamma }}_{0}\left(\xi \right)}{g_{0}\left(\xi \right)}d\xi ,\;\;f_{1}\left(x\right)≔\int_{x}^{x^{*}}\frac{{\bar{\gamma }}_{1}\left(\xi \right)}{g_{1}\left(\xi \right)}d\xi . \end{array}$$ (36)

Further, we use the boundary conditions given in Eqs (27) and (28) to compute the steady-state values of healthy and mutated stem cells, respectively

$$\begin{array}{c} {\bar{C}}_{0}=\frac{\delta_{M_{0}}{\bar{M}}_{0}\left(1+k_{s}\bar{M}\right)}{k_{0}\left(1-m\right)\left[2{\bar{\alpha }}_{D_{0}}+{\bar{\alpha }}_{A_{0}}\right]}e^{f_{0}\left(0\right)} \end{array}$$ (37)

$$\begin{array}{c} {\bar{C}}_{1}=\frac{1+k_{s}\bar{M}}{k_{1}\left[2{\bar{\alpha }}_{D_{1}}+{\bar{\alpha }}_{A_{1}}\right]}[\lambda_{1}e^{f_{1}\left(0\right)}-\frac{m\delta_{M_{0}}{\bar{M}}_{0}}{1-m}e^{f_{0}\left(0\right)}], \end{array}$$ (38)

where $\lambda_{1}≔2m^{\prime }{\bar{\beta }}_{0}\left(0\right){\bar{P}}_{0}\left(0\right)\int_{0}^{x^{*}}e^{f_{1}\left(0\right)}dx-2m^{\prime }\delta_{M_{0}}{\bar{M}}_{0}+\delta_{M_{1}}{\bar{M}}_{1}$. Eventually, we derive the steady-state relation for mutated mature cells ${\bar{M}}_{1}$ from Eq (22):

$$\begin{array}{c} {\bar{M}}_{1}=\frac{1}{\delta_{M_{1}}e^{f_{1}\left(0\right)}}[\frac{\lambda_{2}\delta_{M_{0}}{\bar{M}}_{0}}{\left(1-m\right)}-2m^{\prime }e^{f_{1}\left(0\right)}{\bar{\beta }}_{0}\left(0\right){\bar{P}}_{0}\left(0\right)\int_{0}^{x^{*}}e^{f_{1}\left(0\right)}dx] \end{array}$$ (39)

where $\lambda_{2}=2m^{\prime }\left(1-m\right)e^{f_{1}\left(0\right)}+me^{f_{0}\left(0\right)}-\frac{\gamma_{11}k_{1}\left(2{\bar{\alpha }}_{D_{1}}+{\bar{\alpha }}_{A_{1}}\right)}{\gamma_{10}k_{0}\left(2{\bar{\alpha }}_{D_{0}}+{\bar{\alpha }}_{A_{0}}\right)}e^{f_{0}\left(0\right)}.$ Note that, the steady-state relation for healthy mature cells ${\bar{M}}_{0}$ can be easily determined utilizing Eqs (32) and (39).

From the above derivation of steady-states, we can summarise the following observations. The steady-states of our coupled nonlinear model cannot be defined explicitly, but the sum of the steady-states of healthy and mutated mature cells, used to compute feedback can be represented by an explicit relation. Moreover, the steady-states of stem and progenitor cells highly depend on the steady-state of mature cells due to the feedback inclusion.

### Model simulations

**Fig 3:**
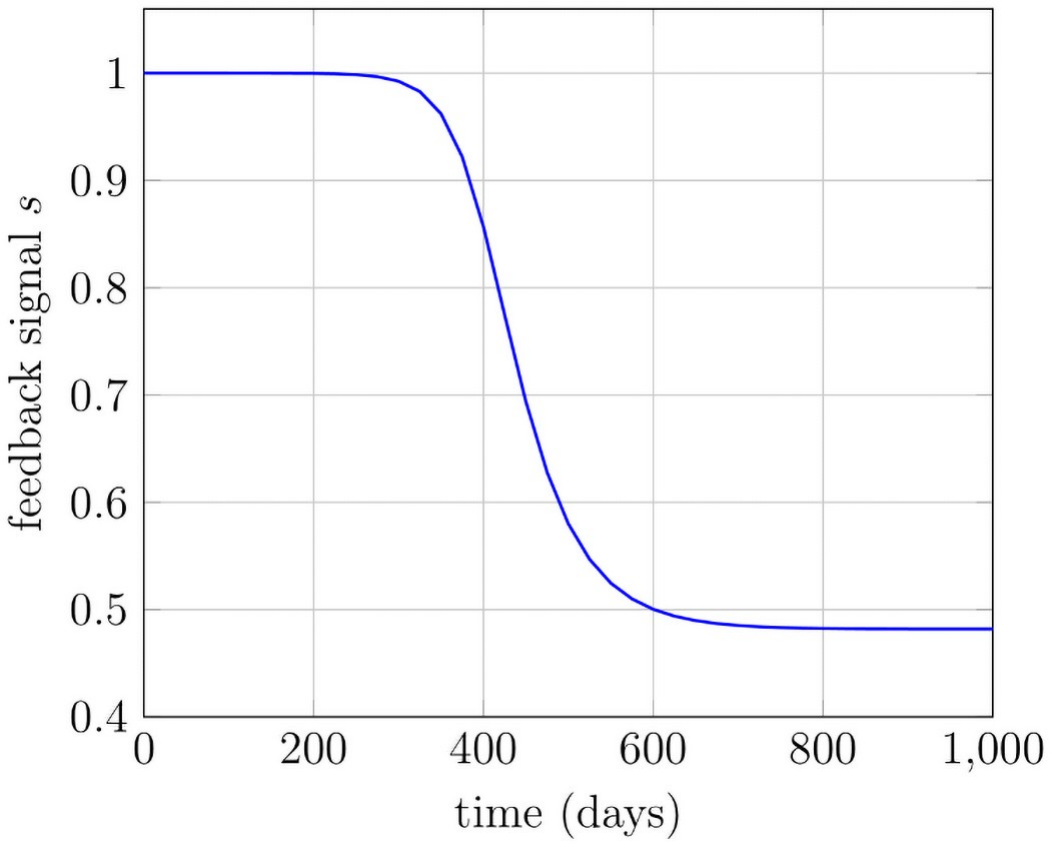
Cytokine feedback signals. With the increasing number of mature healthy and mutated cells, the feedback signal reduces over time.

**Fig 4:**
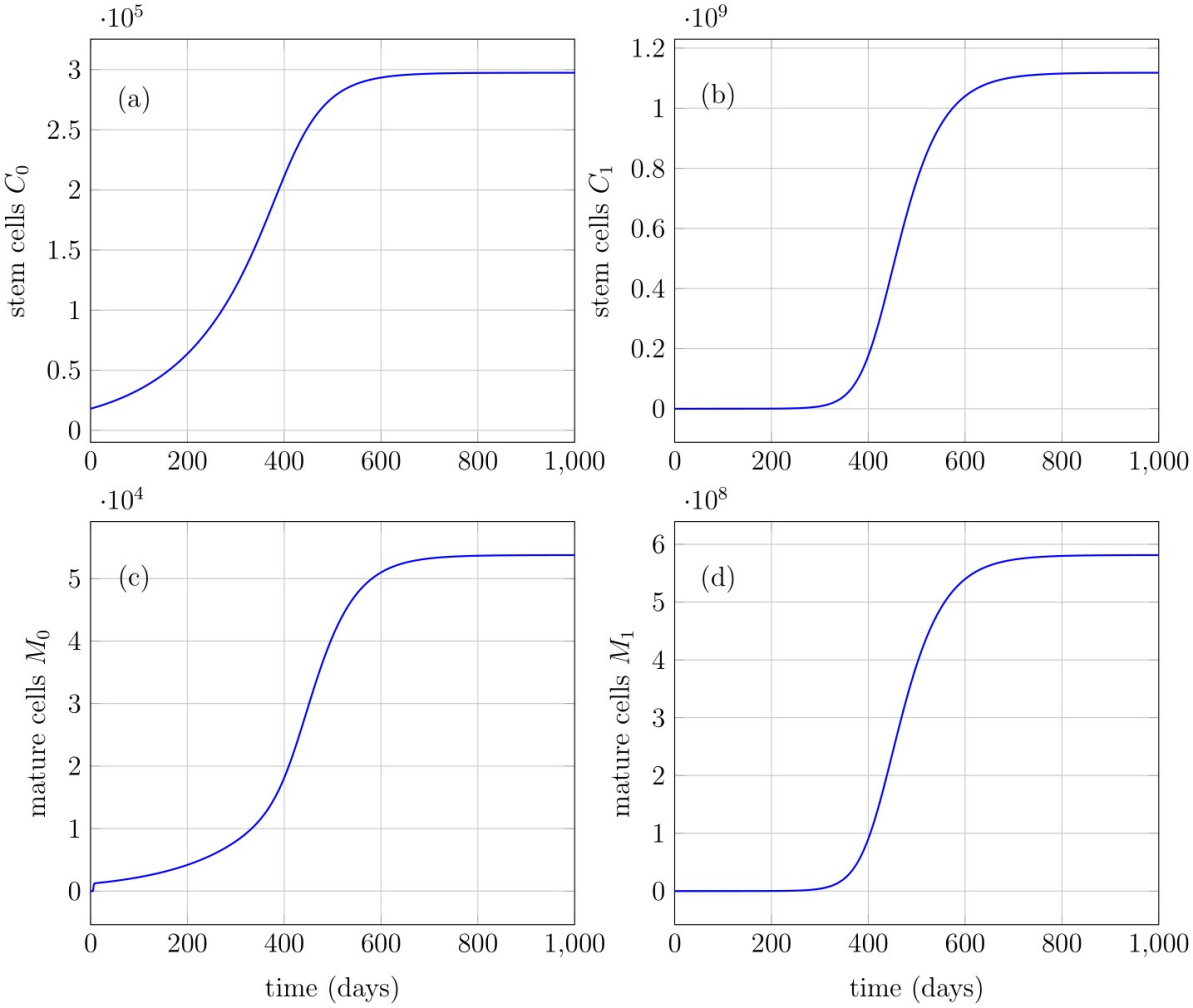
Healthy and mutated stem and mature cells. (a) Healthy stem cells with initial value of 18000*mL*^−1^ grow exponentially and converge to a steady-state. (b) Mutated stem cells with initial condition equal to zero, increase exponentially and attain a steady-state at relatively large value. (c) Healthy mature cells depict a similar behavior with initial condition equal to zero. (d) Mutated mature cells depict a similar behavior with initial condition equal to zero.

**Fig 5:**
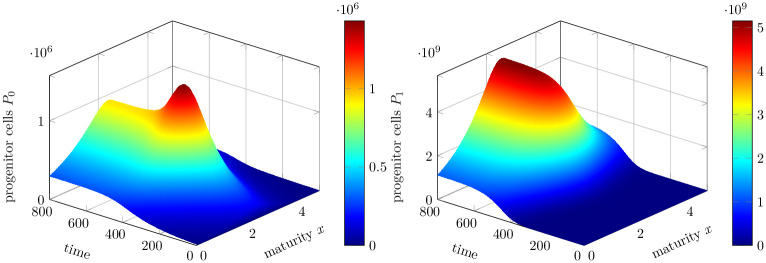
Density distribution of progenitor cells. Left: Distribution of healthy progenitor cells *P*_0_(*t*, *x*). Right: Distribution of mutated progenitor cells *P*_1_(*t*, *x*). It continues to grow towards a higher number and approaches a steady-state.

In this section, we present the model simulations for illustration purposes. The initial states and the used parameters are given in Table 1. The forthcoming results are computed by the numerical scheme given at the end of this section. The maturity variable *x* belongs to [0, 5] with the value of maximum maturity x* set to be 5. The step sizes for time Δ*t* and maturity Δ*x* used in simulations are 0.01 and 0.05, respectively. The behavioral patterns of the model are investigated hereby with the objective to observe the evolution of all six sub-populations with the feedback regulation, which is determined from the total number of both healthy and mutated mature cells. In general, after acquiring a mutation, the mutated cell gains fitness and thus differ considerably from healthy cells [17]. Therefore, the probability of mutated stem cells to self-renew is greater in simulations relative to the healthy ones, while the death rate is reduced, see Table 1. The feedback influences the the probabilities of symmetric/asymmetric self-renewal and differentiation in stem cells whereas in progenitor cells, the feedback is influencing the maximum proliferation rate *b*_*i*_ in the birth function *β*(*x*, *s*). The simulations are initialized with healthy stem cells as *c*_0_ ≔ *C*_0_(0) = 18000*mL*^−1^, while all the rest of the sub-populations have been set to zero. Initially, the feedback signal is maximum, i.e., equal to one, because no differentiated cells exist, while over the course of time, the increase in the healthy and mutated mature cell population leads to a reduction of the feedback signal, as shown in Fig 3. The parameters used in Figs 3–5 are given in Table 1. The exponential growth in healthy stem cell population results in the increase of all healthy and mutated cell types Fig 4(a). A steady-state is achieved in healthy stem cells and consequently in all other sub-populations, see Figs 4 and 5. The achieved steady-states coincide with the analytically calculated steady states of all cell states in Eqs (35)–(39). The feedback signal plays the central role in the stabilization of the model states. In the absence of this feedback signal, the exponential growth continues and thus results in an unnatural number of cells.

**Table 1:** Initial values and parameters of the model for both, healthy and mutated cell lineages, where *i* = 0 stands for the parameters of healthy cell line with zero mutation and *i* = 1 represents the parameters of mutated cell lineage with one mutation.

Parameters and their description	Parameters values		Units
	*i* = 0	*i* = 1	
*c*_*i*_: Initial stem cell density	18000 [19, 43]	0	*mL*^−1^
*m*_*i*_: Initial mature cell density	0	0	*mL*^−1^
${\bar{\alpha }}_{S_{i}}$: Maximum symmetric self-renew probability	0.1846 [44]	0.25	-
${\bar{\alpha }}_{A_{i}}$: Maximum asymmetric self-renew probability	0.6554 [44]	0.60	-
${\bar{\alpha }}_{D_{i}}$: Maximum differentiation probability	0.16 [44]	0.15	-
$\delta_{C_{i}}$: Stem cells’ death rate	0.0125 [19]	0.053 [36]	day^−1^
$\delta_{M_{i}}$: Mature cells’ death rate	1.8 [36]	1.9 [36]	day^−1^
*m*: Mutation rate of stem cells	10^−4^ [45, 46]	-	-
*m*′: Mutation rate of progenitor cells	10^−6^ [47]	-	-
*k*_*i*_: Stem cell proliferation rate	0.47 [31]	0.60	day^−1^
$\omega_{\beta_{i}}$: Maturity at proliferation switch	2.50	2.50	days
$\rho_{\beta_{i}}$: Steepness of progenitor cells proliferation switch	2	2	-
$\omega_{\mu_{i}}$: Maturity at death switch	2.50	2.70	days
$\rho_{\mu_{i}}$: Steepness of progenitor cells death switch	2	2	-
*b*_*i*_: Max. progenitor cells proliferation rate	1.51 [48]	1.8	day^−1^
*d*_*i*_: Max. progenitor cells death rate	2.15	1.8	day^−1^
*k*_*s*_: Ratio of *γ* to *δ*_*S*_	1.85 × 10^−9^	1.85 × 10^−9^	-

**Fig 6:**
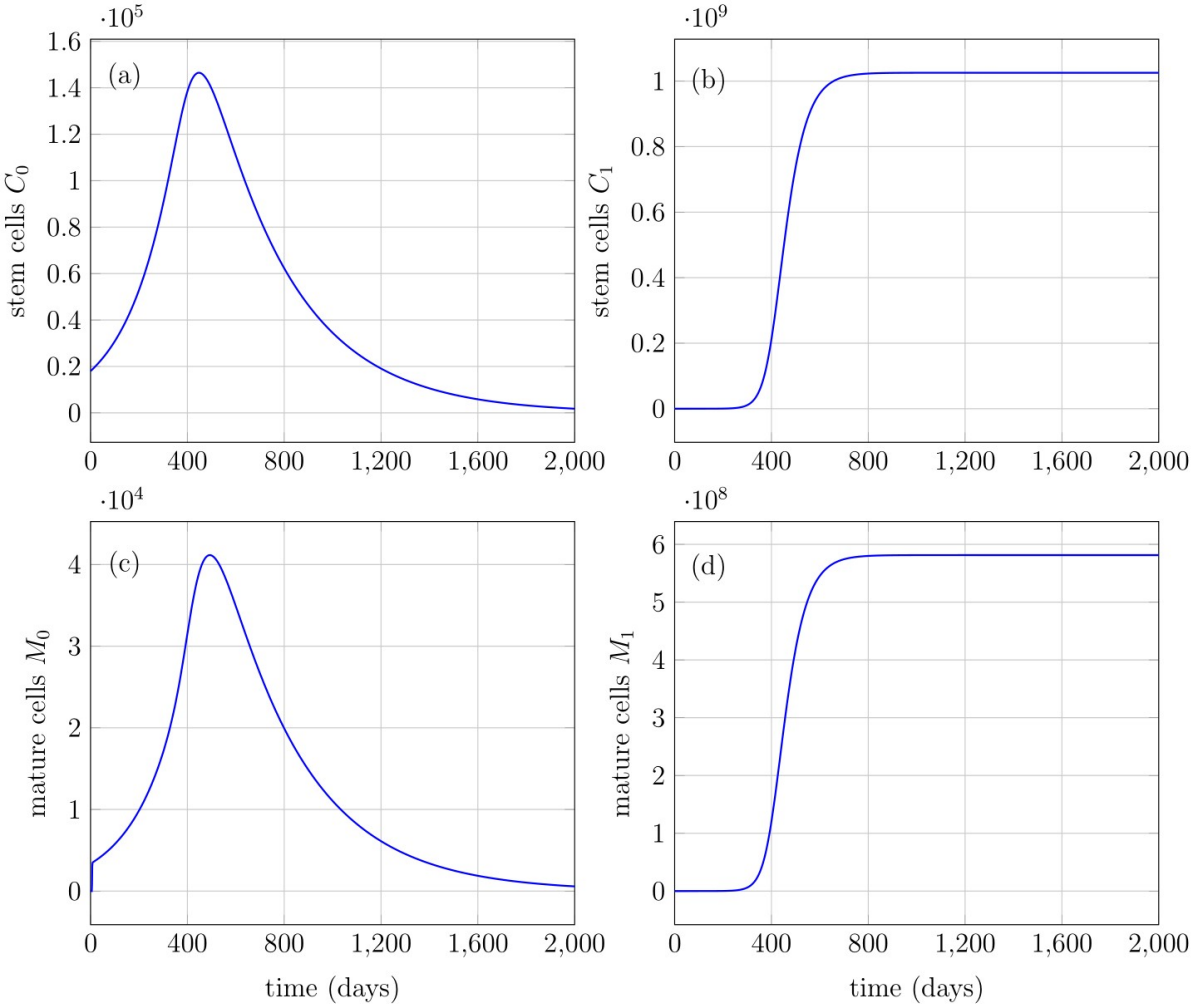
Number of healthy and mutated stem and mature cells. (a) Healthy stem cells with initial value of 18000*mL*^−1^ grow exponentially and start decreasing in number. (b) Mutated stem cells with initial condition equal to zero, increase exponentially until attain a steady-state at relatively large value. (c) Healthy mature cells with initial condition equal to zero, depict the same behavior as healthy stem cells. (d) Mutated mature cells depict a similar behavior as mutated stem cells with initial condition equal to zero.

Fig 6 depicts another behavior of the model in which all the parameters used are same as in Table 1 but the symmetric self-renewal rate of stem cells ${\bar{\alpha }}_{S_{0}}=0.175$ and their death rate $\delta_{C_{0}}=0.016$ day^−1^. Contrary to Fig 4, the healthy stem cells *C*_0_ in Fig 6(a) start decreasing after a gradual increase for a while and eventually land to a very low number. Indeed, similar behavior has been shown by the healthy mature cells in Fig 6(c), whereas the mutated stem and mature cells still attain their respective steady-states, as shown in Fig 6(a) and 6(b). In accordance with the dynamics of healthy and mutated stem cell populations described in Eqs (1) and (2), respectively, the probabilities of symmetric/asymmetric self-renewal and differentiation rates are influenced by the feedback signal. The rapidly increasing healthy and mutated mature cell number in Fig 6(c) and 6(d) tends to abate the value of cytokine feedback signals pursuant to the relation in Eq (12). It can be easily observed from Eq (1) that, as the feedback signal drops, the probabilities of symmetric/asymmetric stem cell self-renewal $\alpha_{S_{0}}/\alpha_{A_{0}}$ and differentiation $\alpha_{D_{0}}$ also decrease. Note that these probabilities vary linearly with feedback signal *s* having a positive slope. As a consequence, with the temporal evolution of healthy stem cells, death rate dominates over self-renewal, and healthy stem cells start declining in number, cf. Eq (1).

In Eq (2) for mutated stem cells, the decline in feedback signal reduces the probability of self-renewal and differentiation in mutated stem cells. Nevertheless, the mutated cells still manage to grow in a higher number due to the increased fitness as stated before and thus approaching a steady-state. This scenario, in which the healthy cell line deteriorates and only mutated cells prevail over time, could also be called ‘pure cancerous steady-state’. The establishment of the steady-state via feedback regulation has already been suggested in the literature [11], where the authors have considered ODE settings for the discrete cell populations of the stem cells all the way to the differentiated cells. Moreover, it is mentioned that the whole dynamics of stem cell lineages can be controlled by a single negative feedback loop, i.e., cytokine signaling.

**Fig 7:**
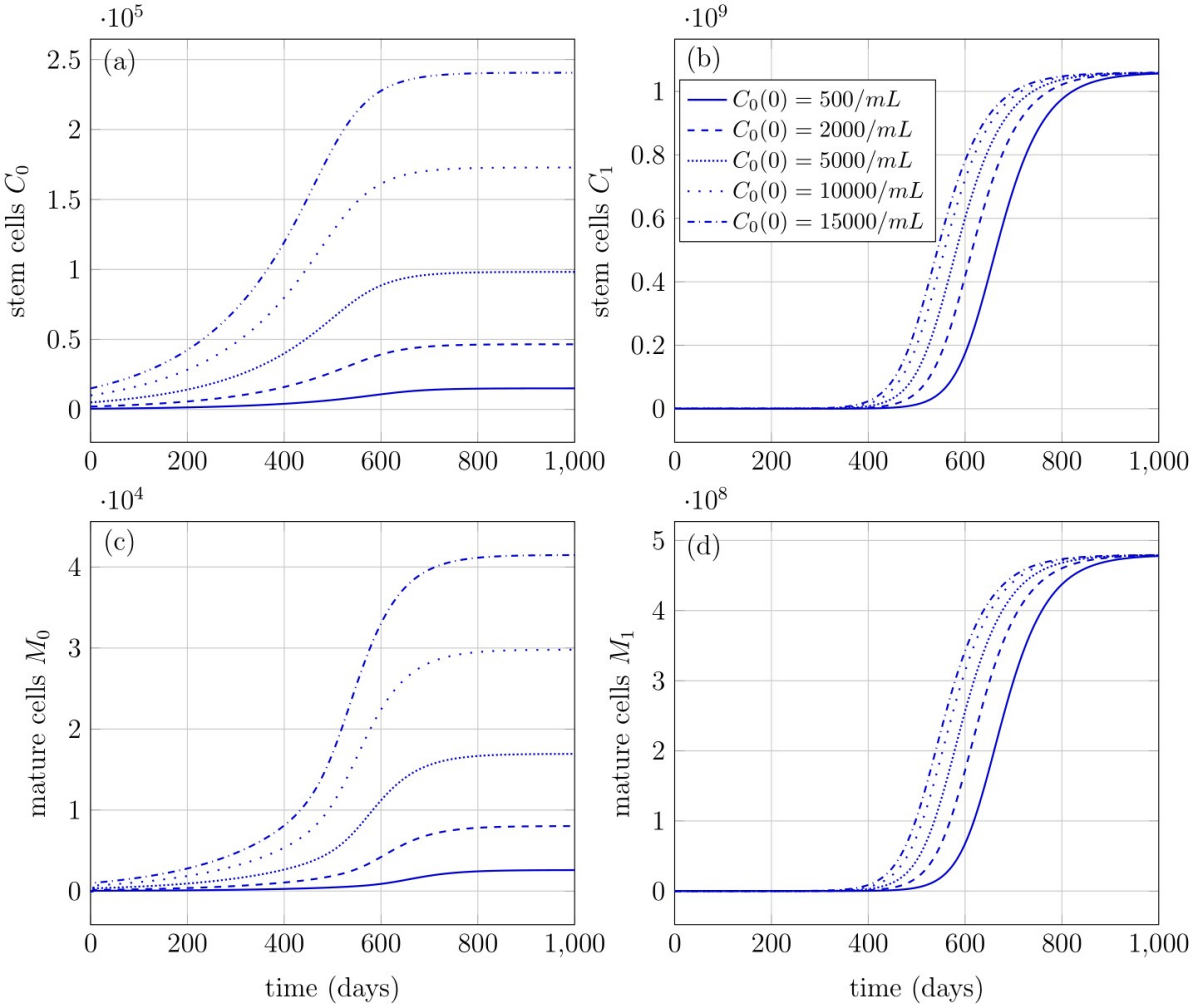
Number of healthy and mutated stem and mature cells with different initial conditions of healthy stem cells. The rest of the states have initial conditions equal to zero. (a) Healthy stem cells achieve a respective steady state for a corresponding initial value. (b) Mutated stem cells with initial condition equal to zero, attain a same steady-state at all initial values of *C*_0_(*t*). (c) Healthy mature cells with initial condition equal to zero, depict the same behavior as healthy stem cells. (d) Mutated mature cells depict a similar behavior as mutated stem cells with initial condition equal to zero.

Fig 7 demonstrates the behavior of the model concerning different initial values of the stem cell population. All other cell population states (mutated stem cells, healthy and mutated progenitor cells, and mature cells) are initialized with zero number of cells. The parameter values used are similar as in Table 1. It can be seen that with any number of initial healthy stem cells *C*_0_(0), the steady states are achieved at the same time in healthy stem *C*_0_(*t*) and mature *M*_0_(*t*) cells, Fig 7(a) and 7(c); however, the magnitudes of the steady states are different. On the other hand, in mutated cell populations, the effect of different initial conditions is reflected only in the rates at which the steady states are achieved, while the magnitude of steady-state is the same for all initial values, see Fig 7(b) and 7(d). It implies that no matter how many healthy stem cells are there at any particular age, the subsequent mutations can lead to a substantial amount of mutated cell populations.

#### Feedback signal as Hill function

**Fig 8:**
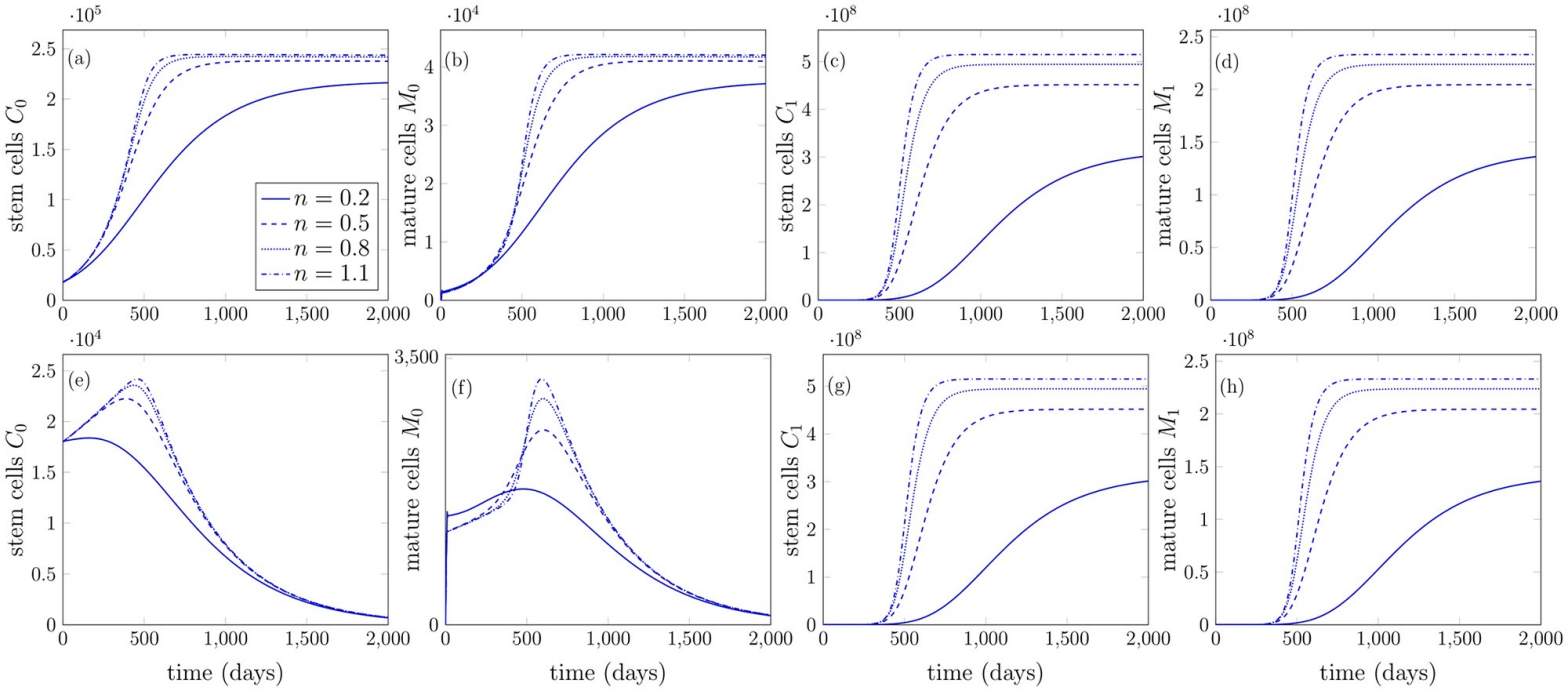
Model behavior by using Hill feedback function for various Hill coefficient values. (a)-(d) All cell states achieve non-trivial steady states. (e)-(h) Pure cancerous steady states where healthy cells decline with time.

Here we want to analyse the model behavior when we define the relation between feedback signal *s* and total number of mature cells *M* using Hill function as compared to the behavior produced by the relation in Eq (12). Since increase in the concentration of mature cells represses the feedback signal, we define their relation using the Hill function as follows:

$$\begin{array}{c} s=\frac{1}{1+(\frac{M}{K_{M}}{)}^{n}}\;, \end{array}$$ (40)

where *K*_*M*_ is the mature cell concentration (2.6 × 10^6^ *mL*^−1^) at which feedback signal is half a maximum and *n* is the Hill coefficient. Note that the Eq (40) coincides with Eq (12) when *n* = 1 and *K*_*M*_ = 1/*k*_*s*_. The simulations have been performed using Hill feedback function in Eq (40) and it turns out that the model depicts the similar behavior to the previous case, compare Fig 8 with Figs 4 and 6. In Fig 8(a)–8(d), the model parameters used are similar as in Table 1, whereas in Fig 8(e)–8(h) the parameter values which have been varied are symmetric and asymmetric self renewal rate of stem cells as ${\bar{\alpha }}_{S0}=0.175$ and ${\bar{\alpha }}_{A0}=0.6650$, respectively.

It is to be noted that the dynamics of the stem and progenitor cells are maintained in homeostasis by inducing feedback only in the self-renewal rates. One can also achieve homeostasis by inducing the feedback in the death rates [49]. However, death rates are kept constant in the current paper; see Table 1. Moreover, in our model, the division rates of stem cell populations {*k*_*i*_, *i* = 0, 1}, are not depending on feedback signal because it has been validated in [11, 50] that an efficient control mechanism underlies the modulation of self-renewal and differentiation rates as compared to the maintenance of proliferation rates in stem cells. It is evident from the simulation results as well that even without feedback regulation in division rates, the mutated stem cell population does achieve a steady-state.

### Model validation

**Fig 9:**
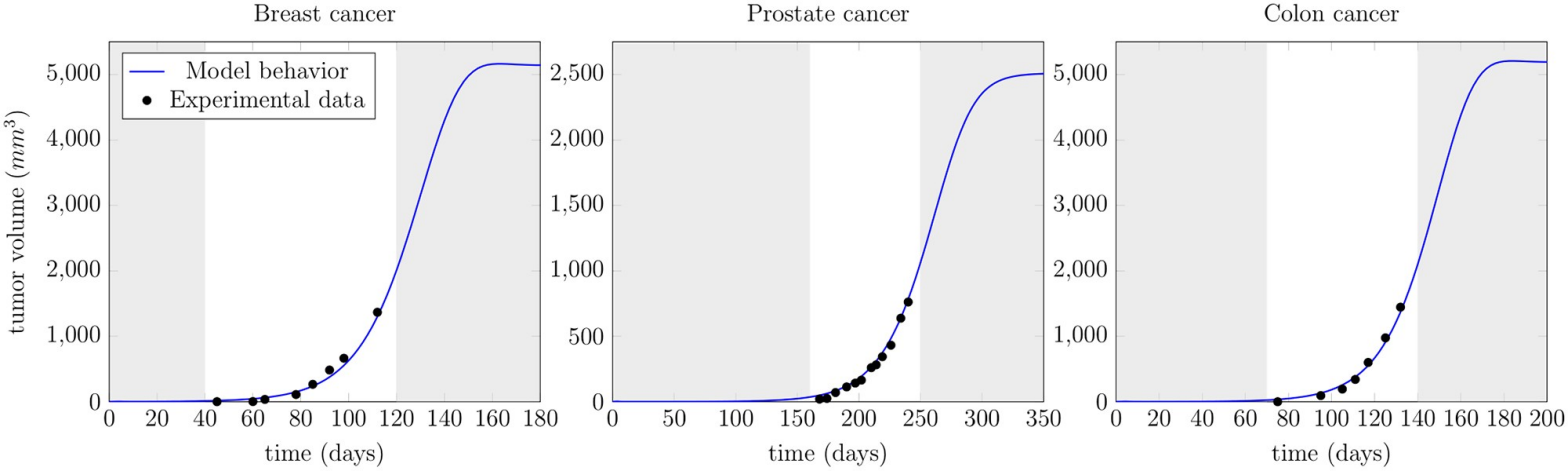
Model fitting to the experimental data. Validation of the model using different experimental data for breast, prostate and colon cancer cells. The data are available only during the exponential growth and the model (blue line) fits the data (black dots) for the given values. The grey shaded regions are the model predictions before and after the available experimental measurements and a steady-state is achieved under cytokine feedback signaling.

In this section, we validate the behavior of our model with different experimental measurements taken from the literature. In Fig 9, we use the tumor volume measurements for three different cancers, namely prostate, breast and colon for validation purpose. The experimental data sets are taken from [51–53]. These data sets are obtained by establishing human tumor xenografts in mice. The only measurements available are tumor volumes from the day of implantation and during exponential growth. We fit our model’s parameters for validation purposes since we do not have the experimentally measured parameter values from these experiments. To compare our model behavior with the tumor volume, we first compute the total number of mutated cells *N*(*t*), which is the sum of mutated stem, progenitor, and mature cells, as

$$\begin{array}{c} N\left(t\right)≔C_{1}\left(t\right)+\int_{x_{0}}^{x^{*}}P_{1}\left(x,t\right)dx+M_{1}\left(t\right). \end{array}$$

Then, considering the effective volume of a cell in the tumor to be 4.18 × 10^−6^ *mm*^3^ [54], the whole tumor volume *V*(*t*) is computed as [55]

$$\begin{array}{c} V\left(t\right)=4.18\times 10^{-6}N\left(t\right)\;mm^{3}. \end{array}$$

The tumor volume calculated from the cell count of the proposed model (blue lines) fits very well to the experimental data (black marks) in all three scenarios, see Fig 9. The grey shaded regions depict the predicted model behavior before and after the available experimental values. Our model attains a steady-state in all three simulations due to the feedback via cytokine signals. Note that, the steady-states may vary in reality for different cancer types and also individually but the proposed model is flexible enough to depict various steady-state scenarios. The healthy cell lineage is considered to be initially at a steady-state. The parameters used in Fig 9 are given in Table 2.

**Table 2:** Parameters used for model validation in Fig 9. The values of the parameters used in the simulations of Fig 9 are presented for all three cancer types.

Param.	Values			Param.	Values
	Breast	Prostate	Colon		Breast/Prostate/Colon
*c*_0_	7 × 10^4^ *mL*^−1^	3 × 10^4^ *mL*^−1^	2.50 × 10^5^ *mL*^−1^	*c*_1_	1.0 × 10^4^ *mL*^−1^
*k*_0_	1	0.985	1.041	*k*_*s*_	12.8 × 10^−10^
*d*_0_	1.50 day^−1^	1.67 day^−1^ [36]	1.50 day^−1^	$\delta_{M_{0}}$	2.1 day^−1^ [36]
*d*_1_	1.33 day^−1^	1.32 day^−1^	1.36 day^−1^	*m*_0_	3.97 × 10^4^ *mL*^−1^
${\bar{\alpha }}_{S_{0}}$	0.355	0.3175	0.362	$\omega_{\beta_{0}}$	1.45 days
${\bar{\alpha }}_{A_{0}}$	0.60	0.5825	0.584	*m*′	10^−6^ [47]
${\bar{\alpha }}_{D_{0}}$	0.045	0.10	0.054	*b*_1_	0.85 day^−1^ [48]
${\bar{\alpha }}_{S_{1}}$	0.40	0.40	0.39	$\rho_{\mu_{0}}=\rho_{\mu_{1}}$	8
${\bar{\alpha }}_{D_{1}}$	0.10	0.10	0.11	*m*	10^−4^ [45, 46]
*k*_1_	0.61 day^−1^ [31]	0.60 day^−1^	0.61 day^−1^	${\bar{\alpha }}_{A_{1}}$	0.50
$\delta_{C_{0}}$	0.24 day^−1^	0.17 day^−1^	0.24 day^−1^	$\omega_{\mu_{0}}$	2.80 days [36]
$\delta_{C_{1}}$	1.0 day^−1^ [56]	1.6 day^−1^	1.0 day^−1^ [56]	$\omega_{\mu_{1}}$	2.95 days
*b*_0_	1.10 day^−1^	1.23 day^−1^ [48]	1.10 day^−1^	$\rho_{\beta_{0}}=\rho_{\beta_{1}}$	2
$\delta_{M_{1}}$	0.3 day^−1^	0.5 day^−1^	0.3 day^−1^	$\omega_{\beta_{1}}$	1.80 days

**Fig 10:**
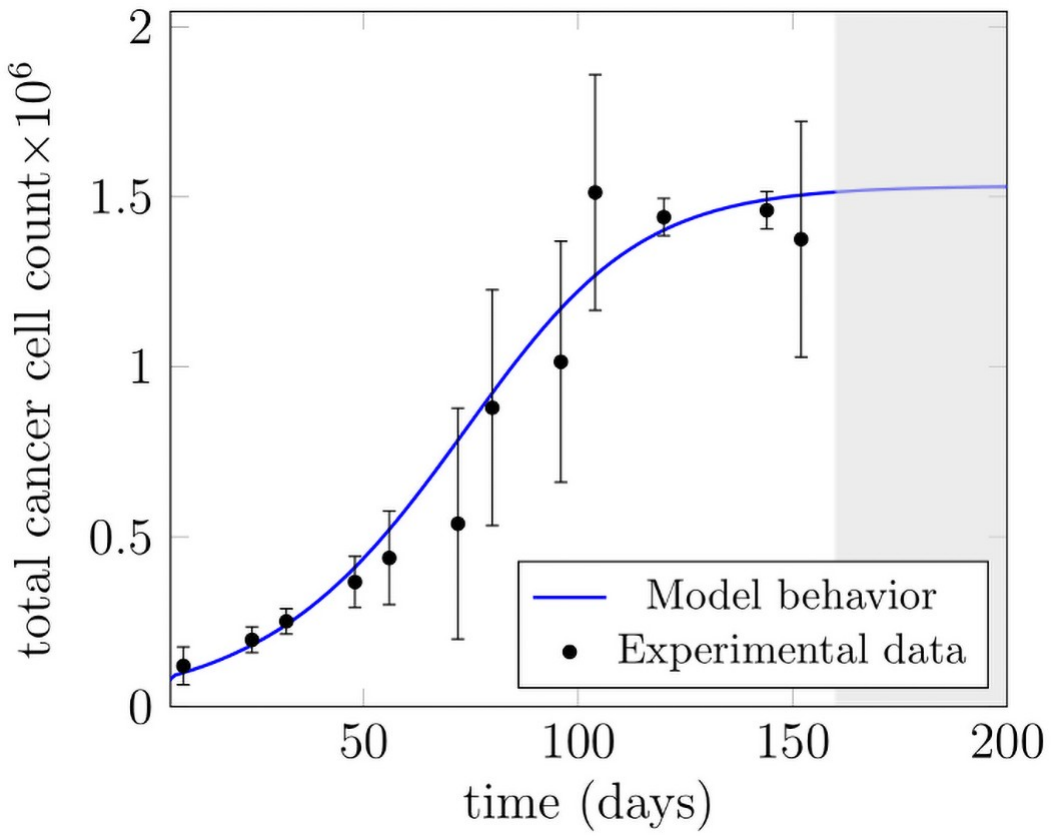
Model fitting to the experimental data. The model has been validated with the data set derived by TUBO Cancer cell line [57]. The experimental data are represented by black error bars representing mean ± standard deviation. The blue line is the model fit to the experimental measurements. Our proposed model predicts an establishment of the steady-state in grey shaded area where no measurements of cell count were available.

The model has been further validated by using another experimental data set generated in vitro experiments on TUBO cancer cells and is reported in [57], see Fig 10. TUBO cancer cells are a cloned line derived in vitro from a BALB-neuT mouse mammary carcinoma. The data set consists of mean ± standard deviation for total cell count. The model fits to the mean values for more than 65 percent of the data set. The initial conditions for healthy cell line are equal to their respective steady-state values. The initial condition of mutated stem cells is equal to 2 × 10^4^ *mL*^−1^ and for mutated progenitor and mature cells are equal to zero. The parameter values used in the Fig 10 which differ from the ones in Table 1 are given in Table 3. The exponential growth and achievement of the steady-state requires enhanced proliferation and self-renewal rates of stem cells. The sum of the probabilities for symmetric self-renewal, asymmetric self-renewal and differentiation is still kept equal to 1.

**Table 3:** Values of the parameters used in Fig 10.

Parameters	Value	Parameter	Value
*m*	0.34 × 10^−5^	${\bar{\alpha }}_{S1}$	0.09
${\bar{\alpha }}_{S0}$	0.3555	${\bar{\alpha }}_{A1}$	0.22
${\bar{\alpha }}_{A0}$	0.6065	${\bar{\alpha }}_{D1}$	0.69
${\bar{\alpha }}_{D0}$	0.038	$\delta_{M_{0}}$	2.20 day^−1^
$\delta_{C_{0}}$	0.2391 day^−1^	$\delta_{M_{1}}$	0.30 day^−1^
$\delta_{C_{1}}$	1.773 day^−1^	*k*_*s*_	1.89 × 10^−7^

### Implemented numerical scheme

In this section, the numerical method used to solve the governing nonlinear Eqs (1)–(10) is presented. We apply already developed finite volume method (FVM) with central upwind scheme for the flux approximation on hyperbolic partial differential equations in MATLAB. The domain of the problem has been discretized in both, space and time. The timeline is discretized into *N*_*t*_ steps with equidistant interval Δ*t* = *t*^*k* + 1^ − *t*^*k*^. The spatial stepsize is given by Δ*x* = x*/*N*_*x*_, where *N*_*x*_ is the maximum number of spatial nodes given by *x*_*j*_ = *j*Δ*x*, 0 ≤ *j* ≤ *N*_*x*_. The discretized progenitor cell density associated with the *j*^th^ spatial interval at time *k* reads

$$\begin{array}{c} P_{i,j}^{k}=\frac{1}{\Delta x}\int_{x_{j-\frac{1}{2}}}^{x_{j+\frac{1}{2}}}P_{i}\left(y,t^{k}\right)dy,\;\text{where}\;\;\;i=0,1. \end{array}$$

The necessary Courant-Friedrichs-Lewy (CFL) condition for convergence of the solution requires ${\text{max}}_{x\in \{x_{j}\}}g_{i}\left(x\right)\frac{\Delta t}{\Delta x}\leq 1.$ The PDEs (3)–(4) are hyperbolic in nature and with the discretization defined above, we can implement the following algorithm to solve the coupled differential equations.

First, the initial conditions are given as

$$\begin{array}{c} C_{i}^{0}=c_{i},\;P_{i,j}^{0}=\frac{1}{\Delta x}\int_{x_{j-\frac{1}{2}}}^{x_{j+\frac{1}{2}}}p_{i}\left(y\right)dy,\;M_{i}^{0}=m_{i},\;\text{for}\;\;i=0,1. \end{array}$$

For each time step *k*, the feedback from mature cells is calculated as

$$\begin{array}{c} s^{k}=\frac{1}{1+k_{s}\left(M_{0}^{k}+M_{1}^{k}\right)}. \end{array}$$

Then, the stem cell population at time *t*^*k*+1^ can be discretized as follows

$$\begin{array}{c} C_{i}^{k+1}\approx C_{i}^{k}+\Delta t(\frac{d}{dt}C_{i}^{k}),\;i=0,1, \end{array}$$ (41)

and the following relation result for healthy and mutated stem cells, respectively

$$\begin{array}{cc} C_{0}^{k+1}= & (1+\Delta t(\left(1-2m\right)\alpha_{S_{0}}\left(s^{k}\right)-m\alpha_{A_{0}}\left(s^{k}\right)-\alpha_{D_{0}}\left(s^{k}\right)-\delta_{S_{0}})k_{0})C_{0}^{k}\\ C_{1}^{k+1}= & (1+\Delta t(\alpha_{S_{1}}\left(s^{k}\right)-\alpha_{D_{1}}\left(s^{k}\right)-\delta_{S_{1}})k_{1})C_{1}^{k}\\ & +\Delta tm(2\alpha_{S_{0}}\left(s^{k}\right)-\alpha_{A_{0}}\left(s^{k}\right))k_{0}C_{0}^{k}. \end{array}$$

The boundary conditions for progenitor cells at *j* = 0 are given as

$$\begin{array}{cc} P_{0,0}^{k+1} & =\left\{\left(1-m\right)\left(2\alpha_{D_{0}}\left(s^{k}\right)+\alpha_{A_{0}}\left(s^{k}\right)\right)k_{0}C_{0}^{k}\right\}/g_{0}\left(x_{0}\right)\\ P_{1,0}^{k+1} & =\left\{\left(2\alpha_{D_{1}}\left(s^{k}\right)+\alpha_{A_{1}}\left(s^{k}\right)\right)k_{1}C_{1}^{k}+\left(2\alpha_{D_{0}}\left(s^{k}\right)+\alpha_{A_{0}}\left(s^{k}\right)\right)mk_{0}C_{0}^{k}\right\}g_{1}\left(x_{0}\right). \end{array}$$

The discretization of the PDEs concerning the density of the progenitor cell populations is given in accordance with the central upwinding scheme as follows

$$\begin{array}{cc} P_{0,j}^{k+1}= & P_{0,j}^{k}-\frac{\Delta t}{\Delta x}(g_{0}\left(x_{j}\right)P_{0,j}^{k}-g_{0}\left(x_{j-1}\right)P_{0,j-1}^{k})\\ & +\Delta t(\left(1-2m^{\prime }\right)\beta_{0}\left(x_{j},s^{k}\right)-\mu_{0}\left(x_{j}\right))P_{0,j}^{k}\\ P_{1,j}^{k+1}= & P_{1,j}^{k}-\frac{\Delta t}{\Delta x}(g_{1}\left(x_{j}\right)P_{1,j}^{k}-g_{1}\left(x_{j-1}\right)P_{1,j-1}^{k})\\ & +\Delta t((\beta_{1}\left(x_{j},s^{k}\right)-\mu_{1}\left(x_{j}\right))P_{1,j}^{k}+2m^{\prime }\beta_{0}\left(x_{j},s^{k}\right)P_{0,j}^{k}). \end{array}$$

Finally, the discretized ODEs for mature cells are given as following

$$\begin{array}{cc} M_{0}^{k+1} & =M_{0}^{k}+\Delta t(g_{0}\left(x_{N_{x}}\right)P_{0,N_{x}}^{k}-\delta_{M_{0}}M_{0}^{k})\\ M_{1}^{k+1} & =M_{1}^{k}+\Delta t(g_{1}\left(x_{N_{x}}\right)P_{1,N_{x}}^{k}+2m^{\prime }\beta_{0}\left(x_{N_{x}},s^{k}\right)P_{0,N_{x}}^{k}-\delta_{M_{1}}M_{1}^{k}), \end{array}$$

which also involves the influx from progenitor cells $P_{0}^{k}$ and $P_{1}^{k}$ at the maximum maturity *x* = x*. The mature cell populations *M*_0_ and *M*_1_ will manipulate the feedback in the next time step and consequently, feedback will alter the dynamics of stem, progenitor or both cell populations to stabilize the exponential growth.

## Discussion

The model predicts the evolution of healthy and mutated stem cell lineages in various case studies. Both lineages evolve with time and achieve a steady state under homeostatic regulation. According to the stem cell hypothesis, the persistence of cancer is regulated by a small number of cancer cells which share the same biological properties as the stem cells [4]. The results of this model are in accordance with the stem cell hypothesis because the mutated stem cells are responsible for the evolution and persistence of the whole mutated cell lineage due to their elevated self-renewal and differentiation potential, see Figs 4 and 5. Moreover, it is comprehensible that an efficient feedback mechanism must exist with heavy cross-talks between the cells themselves and the extracellular environment to robustly regulate the system comprising of cell lineages with various cell types. In the proposed model, the feedback in both the stem and progenitor cell populations, allows mimicking the intercellular interactions among the cells. Besides, the model provides an insight that the self-renewal rate of stem cells is a very sensitive parameter for the persistence and maintenance of healthy cell lines. As shown in Fig 6, the feedback’s influence led to the extinction of the healthy cell line because the self-renewal rate of stem cells was reduced. In a nutshell, the critical ratio of stem cells’ self-renewal rate to the differentiation rate should be preserved to maintain homeostasis in the healthy cell population.

The proposed model has some drawbacks too. First, the model assumes a single mutation leading to cancer evolution, which might not be accurate in many cases, but the model structure is flexible to incorporate more mutations and to predict the evolution of cancer depending on their individual effects. Secondly, the model assumes only cytokines feedback signaling but of course, there exist several feedback mechanisms, for instance, chalone [11], mechanosensing [58] etc. Furthermore, the simulations are performed assuming linear dependence on the feedback signal, which might not be very realistic; however, the precise nature of this feedback is still unknown.

## Conclusion

We propose a generic modeling framework to investigate the coupled dynamics of the healthy and mutated cell lineages, entailing homeostatic regulation. We show that the model predicts familiar behavior and evolutionary patterns of cancer. For instance, the small number of mutated stem cells is responsible for evolving the whole mutated cell lineage. Moreover, the healthy cell line significantly declines in number due to the sensitivity of the symmetric self-renewal rate of stem cells. Thus, the symmetric and asymmetric self-renewal rates of stem cells are crucial for the persistence and maintenance of both cell lines. The model is also validated with different experimental measurements of the tumor available in the literature. Concerning future work, it is possible to extend our model to include additional phenomena, e.g., cell de-differentiation and other feedback mechanisms. Its architecture also enables heterogeneous type mutations to be introduced, which can be of interest to gain additional insights into the development of cancer and, additionally, in the faster emergence of cancer. An appealing future step concerns the stability analysis of the dynamical behavior and sensitivity analysis for the process parameters.

## References

[1] Al-Hajj M, Clarke MF. Self-renewal and solid tumor stem cells. Oncogene. 2004;23(43):7274. 10.1038/sj.onc.1207947 15378087

[2] Fuchs E, Chen T. A matter of life and death: self-renewal in stem cells. EMBO reports. 2013;14(1):39–48. 10.1038/embor.2012.197 23229591 PMC3537149

[3] Elmore S. Apoptosis: a review of programmed cell death. Toxicologic pathology. 2007;35(4):495–516. 10.1080/01926230701320337 17562483 PMC2117903

[4] Reya T, Morrison SJ, Clarke MF, Weissman IL. Stem cells, cancer, and cancer stem cells. Nature. 2001;414(6859):105. 10.1038/35102167 11689955

[5] Hanahan D, Weinberg RA. The hallmarks of cancer. Cell. 2000;100(1):57–70. 10.1016/S0092-8674(00)81683-9 10647931

[6] Jordan CT, Guzman ML, Noble M. Cancer stem cells. New England Journal of Medicine. 2006;355(12):1253–1261. 10.1056/NEJMra061808 16990388

[7] Taylor WR, Fedorka SR, Gad I, Shah R, Alqahtani HD, Koranne R, et al. small-Molecule Ferroptotic Agents with potential to selectively target Cancer stem Cells. Scientific reports. 2019;9(1):5926. 10.1038/s41598-019-42251-5 30976078 PMC6459861

[8] Loeb LA, Loeb KR, Anderson JP. Multiple mutations and cancer. Proceedings of the National Academy of Sciences. 2003;100(3):776–781. 10.1073/pnas.0334858100 12552134 PMC298677

[9] Martincorena I, Raine KM, Gerstung M, Dawson KJ, Haase K, Van Loo P, et al. Universal patterns of selection in cancer and somatic tissues. Cell. 2017;171(5):1029–1041. 10.1016/j.cell.2017.09.042 29056346 PMC5720395

[10] Barrett JC. Mechanisms of multistep carcinogenesis and carcinogen risk assessment. Environmental Health Perspectives. 1993;100:9–20. 10.1289/ehp.931009 8354184 PMC1519586

[11] Lander AD, Gokoffski KK, Wan FY, Nie Q, Calof AL. Cell lineages and the logic of proliferative control. PLoS Biology. 2009;7(1):e1000015. 10.1371/journal.pbio.1000015 19166268 PMC2628408

[12] Johnston MD, Edwards CM, Bodmer WF, Maini PK, Chapman SJ. Mathematical modeling of cell population dynamics in the colonic crypt and in colorectal cancer. Proceedings of the National Academy of Sciences. 2007;104(10):4008–4013. 10.1073/pnas.0611179104 17360468 PMC1820699

[13] Bocharov G, Quiel J, Luzyanina T, Alon H, Chiglintsev E, Chereshnev V, et al. Feedback regulation of proliferation vs. differentiation rates explains the dependence of CD4 T-cell expansion on precursor number. Proceedings of the National Academy of Sciences. 2011;108(8):3318–3323. 10.1073/pnas.1019706108 21292990 PMC3044411

[14] Morrison SJ, Uchida N, Weissman IL. The biology of hematopoietic stem cells. Annual review of cell and developmental biology. 1995;11(1):35–71. 10.1146/annurev.cb.11.110195.000343 8689561

[15] Uchida N, Fleming WH, Alpern EJ, Weissman IL. Heterogeneity of hematopoietic stem cells. Current opinion in immunology. 1993;5(2):177–184. 10.1016/0952-7915(93)90002-A 8099486

[16] Bernard S, Bélair J, Mackey MC. Oscillations in cyclical neutropenia: new evidence based on mathematical modeling. Journal of Theoretical Biology. 2003;223(3):283–298. 10.1016/S0022-5193(03)00090-0 12850449

[17] Ashkenazi R, Gentry SN, Jackson TL. Pathways to tumorigenesis–modeling mutation acquisition in stem cells and their progeny. Neoplasia. 2008;10(11):1170–IN6. 10.1593/neo.08572 18953426 PMC2570593

[18] Colijn C, Mackey MC. A mathematical model of hematopoiesis–I. Periodic chronic myelogenous leukemia. Journal of Theoretical Biology. 2005;237(2):117–132. 10.1016/j.jtbi.2005.03.033 15975596

[19] Michor F, Hughes TP, Iwasa Y, Branford S, Shah NP, Sawyers CL, et al. Dynamics of chronic myeloid leukaemia. Nature. 2005;435(7046):1267. 10.1038/nature03669 15988530

[20] Gentry SN. Mathematical Modeling of Mutation Acquisition in Hierarchical Tissues: Quantification of the Cancer Stem Cell Hypothesis. 2008.

[21] Gentry SN, Jackson TL. A mathematical model of cancer stem cell driven tumor initiation: implications of niche size and loss of homeostatic regulatory mechanisms. PloS one. 2013;8(8):e71128. 10.1371/journal.pone.0071128 23990931 PMC3747196

[22] Weekes SL, Barker B, Bober S, Cisneros K, Cline J, Thompson A, et al. A multicompartment mathematical model of cancer stem cell-driven tumor growth dynamics. Bulletin of Mathematical Biology. 2014;76(7):1762–1782. 10.1007/s11538-014-9976-0 24840956 PMC4140966

[23] Xu S, Kim S, Chen IS, Chou T. Modeling large fluctuations of thousands of clones during hematopoiesis: The role of stem cell self-renewal and bursty progenitor dynamics in rhesus macaque. PLoS computational biology. 2018;14(10):e1006489. 10.1371/journal.pcbi.1006489 30335762 PMC6218102

[24] Sun D, Zhou X, Yu HL, He XX, Guo WX, Xiong WC, et al. Regulation of neural stem cell proliferation and differentiation by Kinesin family member 2a. PloS one. 2017;12(6):e0179047. 10.1371/journal.pone.0179047 28591194 PMC5462413

[25] Velten L, Haas SF, Raffel S, Blaszkiewicz S, Islam S, Hennig BP, et al. Human haematopoietic stem cell lineage commitment is a continuous process. Nature cell biology. 2017;19(4):271–281. 10.1038/ncb3493 28319093 PMC5496982

[26] Dontu G, Al-Hajj M, Abdallah WM, Clarke MF, Wicha MS. Stem cells in normal breast development and breast cancer. Cell proliferation. 2003;36:59–72. 10.1046/j.1365-2184.36.s.1.6.x 14521516 PMC6495427

[27] Berman HK, Gauthier ML, Tlsty TD. Premalignant breast neoplasia: a paradigm of interlesional and intralesional molecular heterogeneity and its biological and clinical ramifications. Cancer prevention research. 2010;3(5):579–587. 10.1158/1940-6207.CAPR-10-0073 20424132

[28] Adimy M, Crauste F, Pujo-Menjouet L. On the stability of a maturity structured model of cellular proliferation. arXiv preprint arXiv:09042492. 2009.

[29] Østby I, Benestad HB, Grøttum P. Mathematical modeling of human granulopoiesis: the possible importance of regulated apoptosis. Mathematical Biosciences. 2003;186(1):1–27. 10.1016/j.mbs.2003.07.003 14527744

[30] Gentry S, Ashkenazi R, Jackson T. A maturity-structured mathematical model of mutation, acquisition in the absence of homeostatic regulation. Mathematical Modelling of Natural Phenomena. 2009;4(3):156–182. 10.1051/mmnp/20094307

[31] Cheshier SH, Morrison SJ, Liao X, Weissman IL. In vivo proliferation and cell cycle kinetics of long-term self-renewing hematopoietic stem cells. Proceedings of the National Academy of Sciences. 1999;96(6):3120–3125. 10.1073/pnas.96.6.3120 10077647 PMC15905

[32] O’Neill A, Schaffer DV. The biology and engineering of stem-cell control. Biotechnology and Applied Biochemistry. 2004;40(1):5–16. 10.1042/BA20030195 15270702

[33] Alison M, Islam S. Attributes of adult stem cells. The Journal of Pathology: A Journal of the Pathological Society of Great Britain and Ireland. 2009;217(2):144–160. 10.1002/path.2498 19085991

[34] Gabriel P, Garbett SP, Quaranta V, Tyson DR, Webb GF. The contribution of age structure to cell population responses to targeted therapeutics. Journal of Theoretical Biology. 2012;311:19–27. 10.1016/j.jtbi.2012.07.001 22796330 PMC3592383

[35] Barreda DR, Hanington PC, Belosevic M. Regulation of myeloid development and function by colony stimulating factors. Developmental & Comparative Immunology. 2004;28(5):509–554. 10.1016/j.dci.2003.09.010 15062647

[36] Bernard S, Bélair J, Mackey MC. Oscillations in cyclical neutropenia: new evidence based on mathematical modeling. Journal of theoretical biology. 2003;223(3):283–298. 10.1016/S0022-5193(03)00090-0 12850449

[37] Rubinow S. A maturity-time representation for cell populations. Biophysical journal. 1968;8(10):1055–1073. 10.1016/S0006-3495(68)86539-7 5679389 PMC1367655

[38] Ullah M, Meziani S, Shah S, Kaci R, Pimpie C, Pocard M, et al. Differentiation of cancer cells upregulates HLA-G and PD-L1. Oncology Reports. 2020;43(6):1797–1804. 10.3892/or.2020.7572 32236615 PMC7160553

[39] Lakshmanaswamy R. Approaches to understanding breast cancer. Academic Press; 2017.

[40] Goldring MB, Goldring S. Cytokines and cell growth control. Critical reviews in eukaryotic gene expression. 1991;1(4):301–326. 1802112

[41] Metcalf D. Hematopoietic Cytokines. Blood. 2008;111(2):485–491. 10.1182/blood-2007-03-079681 18182579 PMC2200848

[42] Layton JE, Hockman H, Sheridan WP, Morstyn G. Evidence for a novel in vivo control mechanism of granulopoiesis: mature cell-related control of a regulatory growth factor. Blood. 1989;74(4):1303–1307. 10.1182/blood.V74.4.1303.1303 2475185

[43] Abkowitz JL, Catlin SN, McCallie MT, Guttorp P. Evidence that the number of hematopoietic stem cells per animal is conserved in mammals. Blood. 2002;100(7):2665–2667. 10.1182/blood-2002-03-0822 12239184

[44] Wu M, Kwon HY, Rattis F, Blum J, Zhao C, Ashkenazi R, et al. Imaging hematopoietic precursor division in real time. Cell Stem Cell. 2007;1(5):541–554. 10.1016/j.stem.2007.08.009 18345353 PMC2267762

[45] Araten DJ, Golde DW, Zhang RH, Thaler HT, Gargiulo L, Notaro R, et al. A quantitative measurement of the human somatic mutation rate. Cancer Research. 2005;65(18):8111–8117. 10.1158/0008-5472.CAN-04-1198 16166284

[46] Jackson AL, Loeb LA. The mutation rate and cancer. Genetics. 1998;148(4):1483–1490. 10.1093/genetics/148.4.1483 9560368 PMC1460096

[47] Rimoin DL, Connor JM, Pyeritz RE, Korf BR. Emery and Rimoin’s principles and practice of medical genetics. Churchill Livingstone Elsevier; 2007.

[48] Reizenstein P. Growth of normal and malignant bone marrow cells. Leukemia research. 1990;14(8):679–681. 10.1016/0145-2126(90)90093-O 2201819

[49] Aglietta M, Piacibello W, Sanavio F, Stacchini A, Apra F, Schena M, et al. Kinetics of human hemopoietic cells after in vivo administration of granulocyte-macrophage colony-stimulating factor. The Journal of Clinical Investigation. 1989;83(2):551–557. 10.1172/JCI113917 2643633 PMC303714

[50] Lo WC, Chou CS, Gokoffski KK, Wan FYM, Lander AD, Calof AL, et al. Feedback regulation in multistage cell lineages. Mathematical Biosciences and Engineering. 2009;6(1):59. 10.3934/mbe.2009.6.59 19292508 PMC2756546

[51] Ginestier C, Hur MH, Charafe-Jauffret E, Monville F, Dutcher J, Brown M, et al. ALDH1 is a marker of normal and malignant human mammary stem cells and a predictor of poor clinical outcome. Cell stem cell. 2007;1(5):555–567. 10.1016/j.stem.2007.08.014 18371393 PMC2423808

[52] Ellis WJ, Vessella RL, Buhler KR, Bladou F, True LD, Bigler SA, et al. Characterization of a novel androgen-sensitive, prostate-specific antigen-producing prostatic carcinoma xenograft: LuCaP 23. Clinical Cancer Research. 1996;2(6):1039–1048. 9816265

[53] Ricci-Vitiani L, Lombardi DG, Pilozzi E, Biffoni M, Todaro M, Peschle C, et al. Identification and expansion of human colon-cancer-initiating cells. Nature. 2007;445(7123):111–115. 10.1038/nature05384 17122771

[54] Darnell J, Lodish H, Baltimore D. Molecular cell biology. QH581. 2 D22 1990; 1990.

[55] Molina-Peña R, Álvarez MM. A simple mathematical model based on the cancer stem cell hypothesis suggests kinetic commonalities in solid tumor growth. PloS one. 2012;7(2):e26233. 10.1371/journal.pone.0026233 22363395 PMC3281810

[56] Rodriguez-Brenes IA, Kurtova AV, Lin C, Lee YC, Xiao J, Mims M, et al. Cellular hierarchy as a determinant of tumor sensitivity to chemotherapy. Cancer research. 2017;77(9):2231–2241. 10.1158/0008-5472.CAN-16-2434 28235762 PMC5487257

[57] Chivassa G, Fornari C, Sirovichr R, Pennisi M, Beccuti M, Cordero F. A mathematical model to study breast cancer growth. In: 2017 IEEE International Conference on Bioinformatics and Biomedicine (BIBM). IEEE; 2017. p. 1438–1445.

[58] Vining KH, Mooney DJ. Mechanical forces direct stem cell behaviour in development and regeneration. Nature reviews Molecular cell biology. 2017;18(12):728–742. 10.1038/nrm.2017.108 29115301 PMC5803560

